# Characterization and comprehensive evaluation of phenotypic characters in wild *Camellia oleifera* germplasm for conservation and breeding

**DOI:** 10.3389/fpls.2023.1052890

**Published:** 2023-03-21

**Authors:** Tao Chen, Li Liu, Yiling Zhou, Qian Zheng, Siyuan Luo, Tingting Xiang, Lijun Zhou, Shiling Feng, Hongyu Yang, Chunbang Ding

**Affiliations:** College of Life Science, Sichuan Agricultural University, Ya’an, China

**Keywords:** *Camellia oleifera*, phenotypic characters, DUS testing, genetic diversity, comprehensive evaluation

## Abstract

*Camellia oleifera* Abel. is an economically important woody oil plant native to China. To explore the genetic diversity of wild *C. oleifera* phenotypic traits and effectively protect these germplasm resources, this study provides a thorough evaluation of the phenotypic variability of a cluster of 143 wild *C. oleifera* germplasm resources. A total of 41 characters, including leaves, flowers, fruits, seeds, and oil quality characters, were investigated based on the quantization of physical and chemical descriptors and digital image analysis. The findings revealed significant variations among the 41 characters with a high range of Shannon–Wiener indexes (*H*′) from 0.07 to 2.19. The coefficient of variation (CV) among 32 quantitative characters ranged from 5.34% to 81.31%, with an average of 27.14%. High genetic diversity was also detected among the 143 germplasm. Based on the analysis of hierarchical clustering, 143 accessions were separated into six categories. All the individuals can be clearly distinguished from each other according to the result of the principal component analysis (PCA). The M-TOPSIS exhaustive evaluation method based on correlation and PCA analyses of 32 quantitative characters was applied for the 143 wild *C. oleifera* accessions, and the top 10 varieties were identified as YA53, YA13, YA40, YA34, YA57, YA19, YA33, YA41, DZ8, and YA7. This research optimized the germplasm evaluation system and perfected the statistical phenotypic traits for distinctness, uniformity, and stability (DUS) testing. Some top-notch germplasm sources were also screened for oil-tea *Camellia* breeding.

## 1 Introduction

*Camellia oleifera* Abel. is a small evergreen tree or shrub belonging to the family Theaceae, genus *Camellia.* Generally, oil tea (*C. oleifera*) in consort with olive (*Olea europaea* L.), oil palm (*Elaeis guineensis* Jacq.), and coconut (*Cocos nucifera* L.) that produce edible oil are regarded as the four famous woody oil crops in the world (Xiao et al., 2017; Luan et al., 2020). The oil is extracted from *C. oleifera* seeds and used as both traditional Chinese medicine and a nutrient-rich edible oil with a transparent hue, high purity level, and abundant nutrients (Lee and Yen, 2006). It is rich in monounsaturated fatty acids, sterols, squalene, vitamin E, polyphenols, and other bioactive compounds, known as “eastern olive oil” (Mahboubifar et al., 2016; Xiao et al., 2017). Numerous studies demonstrated that *C. oleifera* oil is effective against ulceration, oxidation, and inflammation, and that long-term consumption can reduce cholesterol, blood pressure, and blood lipid levels; delay atherosclerosis; and prevent the deterioration of neurological function (Bumrungpert et al., 2016; Jung et al., 2019). However, due to a steady rise in population, *C. oleifera* oil production is significantly lower than demand, and it is challenging to maintain a balance between demand and supply. Consequently, there is an undeniable requirement to enhance *C. oleifera* cultivation.

Rich in genetic variation, the wild *C. oleifera* germplasm resources are potential resources containing vast quantities of superior traits for breeding (Cheng et al., 2018). Phenotypic diversity is a comprehensive manifestation of the interaction between biological genetics and environmental factors, which is one of the primary research foci in genetic diversity (Costa et al., 2017). Detecting genetic variation from phenotypic traits can reveal the extent of genetic variation to a certain degree. Assessing the phenotypic variation of different individuals will benefit the protection and utilization of germplasm (Zhou et al., 2015). Moreover, qualitative traits can facilitate the selection of resources for the genetic enhancement of *C. oleifera*. In general, determining the level of phenotypic genetic diversity in wild resources can reduce the redundancy of germplasm resource protection, promote the development of core germplasm resources, and facilitate the efficient utilization of genetic resources in breeding (Verma et al., 2019).

The standardized description and evaluation of distinctness, uniformity, and stability (DUS) for the traits, including plants, shoots, buds, leaves, flowers, fruits, seeds, and phenological characteristics, are the basis for the grant of protection to new plant varieties by the International Union for the Protection of New Varieties of Plants (UPOV) (TG/275/1). In this study, 41 phenotypic characters of 143 wild *C. oleifera* germplasm resources were characterized and comprehensively evaluated. The aim was to evaluate the phenotypic diversity and variation of leaves, flowers, fruits, seeds, and oil quality characters, rank these investigated phenotypic characters using statistical analysis, and optimize the phenotypic investigation standard of the oil-tea *Camellia* DUS test. The results of this study will be greatly helpful for the protection of wild *C. oleifera* resources and the screening of potential germplasms with excellent traits. It also provides a reference for the further utilization of *C. oleifera* and the genetic improvement of main characters, as well as a theoretical basis for the breeding of new varieties in the future.

## 2 Materials and methods

### 2.1 Plant materials and experimental sites

**Figure 1:**
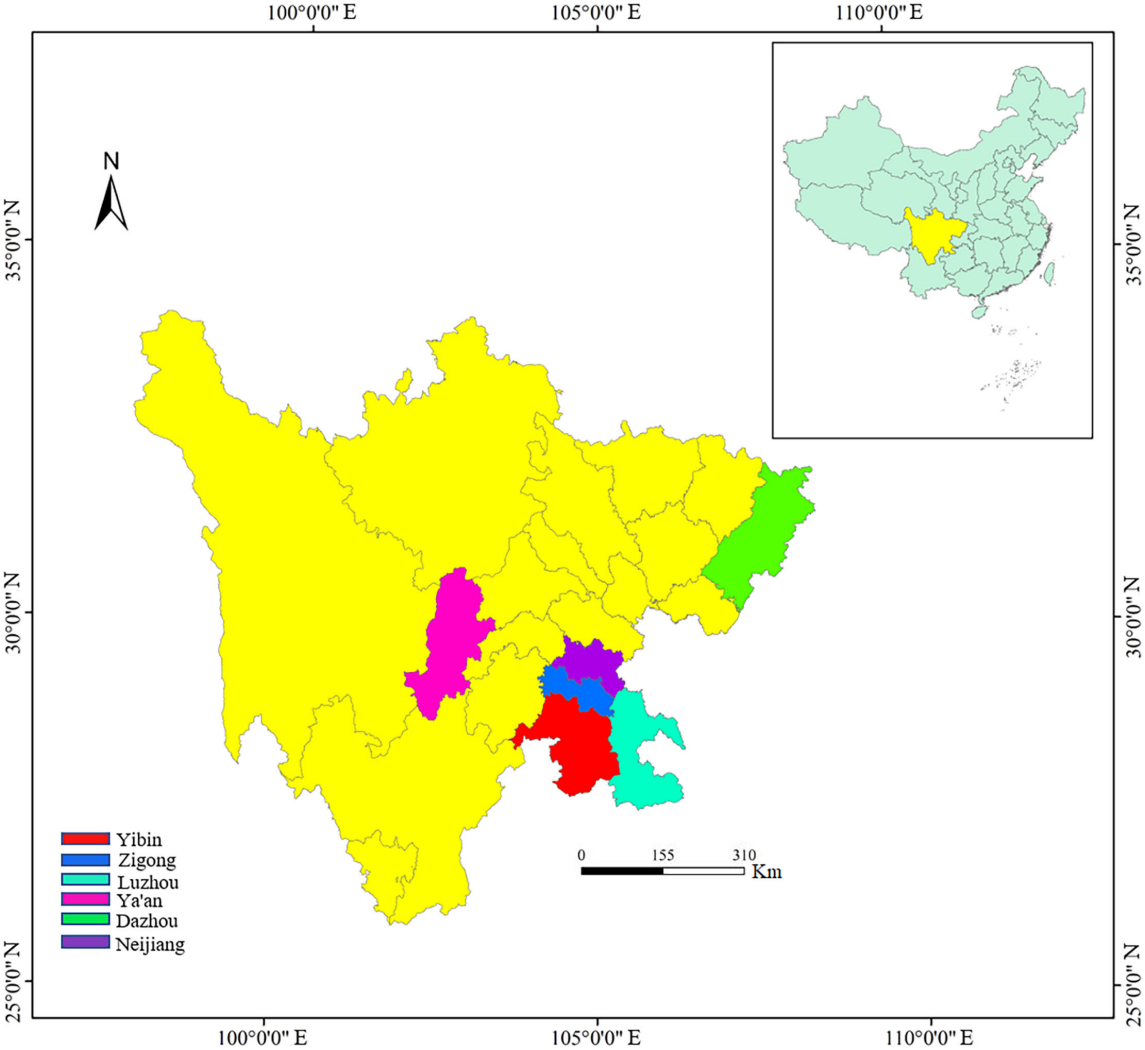
The city distributing the collection of wild *C. oleifera* germplasm resources.

In the past 5 years, we have investigated the distribution and conservation status of wild *C. oleifera* in the wide regions of southwest China (Sichuan province). Some individuals with excellent traits and distinct morphological variation were selected and marked in the field. A total of 143 wild accessions were finally clustered and used in this study, including Ya’an (YA, 60), Zigong (ZG, 16), Yibin (YB, 18), Luzhou (LZ, 7), Dazhou (DZ, 9), and Neijiang (NJ, 33) (**Figure 1**). All the germplasms were more than 10 years old growing naturally in the wild without fertilization and management. We performed a field survey of these resources for 2 consecutive years (2020 and 2021) and collected leaves, flowers, fruits, and seeds for the measurement of morphological traits.

### 2.2 Measurement of phenotypic traits

At the stage of fruit physiological ripening, 30 healthy mature leaves, blooming flowers, and disease-free mature fruits per tree were randomly collected around the outer part of the canopy. For the determination of fatty acids and chemical composition, fresh fruits were picked from trees, saved in ice boxes, and rapidly transferred to the laboratory, where they were stored at 4°C.

#### 2.2.1 Measurement of appearance (phenotypic character)

Data for one qualitative character (relative height of pistil and stamen) and eight pseudo-qualitative characters (leaf shape, shape of leaf apex, petal color, stamen variation, fruit shape, peel color, seed color, and seed shape) were determined according to the guidelines provided by the State Forestry Administration of the People’s Republic of China (LY/T 2742-2016) and previous research (Peng et al., 2007) with some modifications (**Supplementary Table S1**).

For quantitative traits, the leaf length, leaf width, diameter of a flower crown, petal length, fruit height, fruit diameter, and peel thickness were measured by a vernier caliper with 0.01 mm precision. The number of petals, sepals, stigmas, and seeds was measured by visual observation. Fruit weight was evaluated by an electronic balance with 0.01 g precision. The index of leaf size and shape were calculated as follows: leaf area = 2/3 leaf length × leaf width; leaf shape index = leaf length/width, shape index of fruit = fruit height/diameter; and area of flower crown = transverse diameter × longitudinal diameter.

Some indexes of economic characters were measured and calculated according to the previous study (You et al., 2019), the fresh seed rate = (fresh seed weight/fresh fruit weight) × 100%, dry seed rate = (dry seed weight/fresh fruit weight) × 100%, kernel rate of dry seed = (dry kernel weight/dry seed rate) × 100%, moisture rate of fresh seed = (fresh seed weight − dry seed weight)/fresh seed weight × 100%, oil rate of kernel = (oil weight/kernel weight) × 100%, oil rate of dry seed = oil rate of kernel × kernel rate of dry seed × 100%, and oil rate of fresh fruit = oil rate of kernel × dry seed rate × 100%.

#### 2.2.2 Investigation of oil and intrinsic quality traits

The oil of *C. oleifera* was obtained according to the national standard of China (GB/T 14488.1-2008). In short, the Soxhlet extraction method was performed with petroleum ether as the extraction solvent. The extraction temperature was 85°C and lasted 8 h. The oil was stored in the dark at 4°C for the following analysis. Each sample was repeated three times.

The measurement of acid value and peroxide value for *C. oleifera* oil was determined according to national standards (GB 5009.229-2016 and GB5009.227-2016, respectively).

The fatty acid composition was established by gas chromatography-mass spectrometry (GC-MS) after transesterification (Liu et al., 2021). In total, 100 mg of oil was treated with 2 ml of 1 mol/L NaOH-methanol. The sample was mixed on a vortex mixer and shaken for 30 min at 40°C. The methyl esters were extracted with 2 ml *n*-hexane. Then, the GC-MS analysis was conducted by Agilent 7890A gas chromatograph and 5977C mass spectrometry (Agilent Technologies, Palo Alto, California, USA) and equipped with a capillary column HP-5MS (30 m × 0.25 mm; 0.25 µm). The oven temperature was programmed from 60°C for 2 min, increasing at 15°C/min to 150°C holding for 2 min, then 15°C/min increasing to 280°C for 3 min. The carrier gas was helium, with a flow rate of 0.6 ml/min. The injector temperature was 240°C, and the detector temperature was 260°C. The mass scans ranged from 50 to 500 m/z.

The content of tocopherol was determined according to the previous method (Cao et al., 2015). A sample containing 1 g of oil was dissolved in *n*-hexane at a fixed volume of 10 ml, mixed, and filtered by a 0.22-μm microporous membrane for HPLC (Agilent Technologies, Palo Alto, California, USA) equipped with a ZORBAX SB-C18 column (150 mm × 4.6 mm, 5.0 µm)) analysis. The detection conditions were listed as follows: a fluorescence detector, the excitation wavelength was 295 nm and the emission wavelength was 325 nm, the mobile phase was methanol at a flow rate of 0.8 ml/min, and the column temperature was 35°C.

The content of squalene was evaluated according to the previous method with some modifications (Liu, 2017). Firstly, the oil was saponified by a potassium hydroxide-ethanol solution, and then the sample was analyzed by HPLC. The detection conditions were set as follows: an ultraviolet detector, the wavelength was 325 nm; the column temperature was 30°C; and the mobile phase was methanol:acetonitrile (60:40, v:v) at a flow rate of 1.0 ml/min.

The content of total sterols was determined using the method described by Liu et al. (2020). The sample containing 0.2 g of oil was dissolved in 2 ml of a 2.5-mol/L KOH-ethanol solution and shaken for 30 s. Saponification was completed in an 80°C water bath for 1 h, shaking every 10 min. After 1 h, the supernatant was collected and cooled to room temperature. A total of 2.0 ml of deionized water and 5.0 ml of *n*-hexane were added. The supernatant was taken to a 50-ml centrifuge tube. The water layer was extracted twice with *n*-hexane, and 5 ml of *n*-hexane was used each time. The *n*-hexane was volatilized in the water bath to 5 ml, shaken well, washed with deionized water to neutralize it, and transferred to a 10-ml centrifuge tube from the upper *n*-hexane phase. A 1 g of anhydrous sodium sulfate was added to remove water. After standing for 1 min, the sample was then filtered through a 0.45-μm microporous membrane for further analysis. A 0.4-ml sterol extract was mixed with 0.4 ml of the sulfate-phosphate-ferric agent. The absorbance was measured at 480 nm after 30 min of shaking and cooling.

### 2.3 Statistical analysis

All experiments were performed in triplicate. The mean of each trait based on a 2-year investigation and measurement was used for statistical analysis. For qualitative and pseudo-qualitative characters, traits were classified into 10 grades, 1 grade< *X* − 2*σ*, 10 grades > *X* + 2*σ*; each grade interval is 0.5*σ* between 1 and 10 grades; *X* and *σ* are the mean and standard deviation, respectively. The morphological diversity was evaluated by the frequency of trait dispersion and Shannon’s diversity index (*H*′). The statistics of quantitative parameters were measured, including minimum (Min), maximum (Max), mean, median, standard deviation (SD), coefficient of variation (CV, %), and *H*′. The *H*′ for each trait was calculated by using the following formula: H" = − Pi × ln (Pi) (*Pi* is the proportion of the individual number of this trait in total individual number) (Lei et al., 2018). The CV for all quantitative traits was calculated as CV = S /x , where *S* is the standard deviation and $\bar{\text{x}}$ is the mean (Das and Divakara, 2011). The IBM SPSS Statistics version 20.0 (SPSS Inc., Chicago, IL, USA) was performed to estimate correlation among all quantitative traits with the Pearson correlation coefficient. Principal component analysis (PCA) was also applied to determine the relationship among the individuals. OriginPro 9.1 (OriginLab, Northampton, Massachusetts, USA) was used to perform cluster analysis. Correlation and bivariate correlation analyses were calculated by omicshare tools. (https://www.omicshare.com/tools/Home/Soft/getsoft). M-TOPSIS was achieved by MatLab 16.0 (MathWorks Inc., Natick, Massachusetts, USA).

## 3 Results

### 3.1 Leaf and flower phenotypic traits

**Figure 2:**
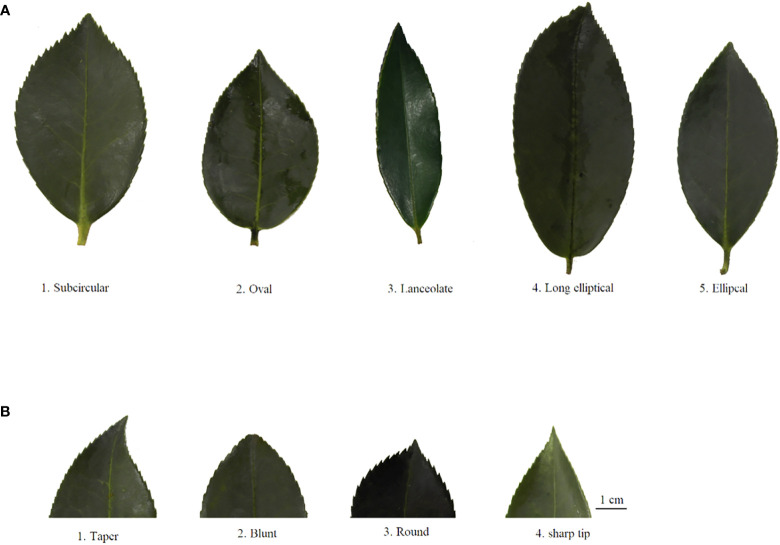
Rating of leaf phenotypic traits for wild *C*. *oleifera* germplasm resources: **(A)** leaf shape and **(B)** shape of leaf apex.

According to the description of the oil-tea *Camellia* DUS test guidelines (LY/T 2742-2016), the leaf shape was classified into four ratings (subcircular, elliptical, long elliptical, and lanceolate). Nevertheless, based on the findings of our study (**Figure 2A**; **Table 1**), the leaf shape of the detected 143 accessions should be classified into five categories: subcircular (16.08%), oval (11.89%), lanceolate (6.29%), long elliptical (11.19%), and elliptical (54.55%). In this study, the shape of the leaf apex was classified into four categories: taper (53.85%), blunt (15.38%), round (6.99%), and sharp (23.78%) (**Figure 2B**; **Table 1**). As shown in **Table 1**, the *H*′ values for the leaf shape and apex shape were 1.31 and 1.15, respectively. For the quantitative characteristics of leaves, the CV values ranged from 11.16% to 24.86%, and the leaf area ranged from 669.34 to 2,320.00 mm^2^ with the highest CV. The *H*′ of leaf quantitative traits varied between 1.99 and 2.11 and also showed high phenotypic diversity (**Table 2**).

**Table 1:** Variability and genetic diversity of qualitative and pseudo-qualitative characters.

Characters	Frequency distribution (%)									*H′*
	1	2	3	4	5	6	7	8	9	
Qualitative characters										
Relative height of pistil and stamen	36.36	20.98	42.66							1.06
Pseudo-qualitative characters										
Leaf shape	16.08	11.89	6.29	11.19	54.55					1.31
Shape of leaf apex	53.85	15.38	6.99	23.78						1.15
Petal color	98.60	1.40								0.07
Stamen variation	98.60	1.40								0.07
Fruit shape	4.90	0.70	10.49	1.40	9.09	12.59	52.45	8.39		1.50
Peel color	1.40	22.38	3.50	47.55	24.48	0.70				1.59
Seed color	23.08	26.57	47.55	2.80						1.14
Seed shape	14.69	20.98	4.90	41.96	17.48					1.43

**Figure 3:**
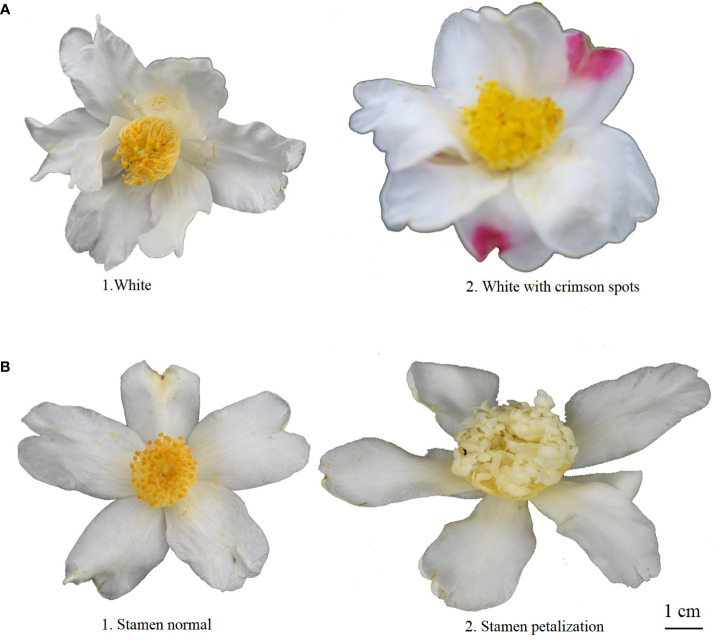
Rating of flower morphologic characters for wild C. oleifera germplasm resources: **(A)** petal color and **(B)** stamen variation.

**Figure 4:**
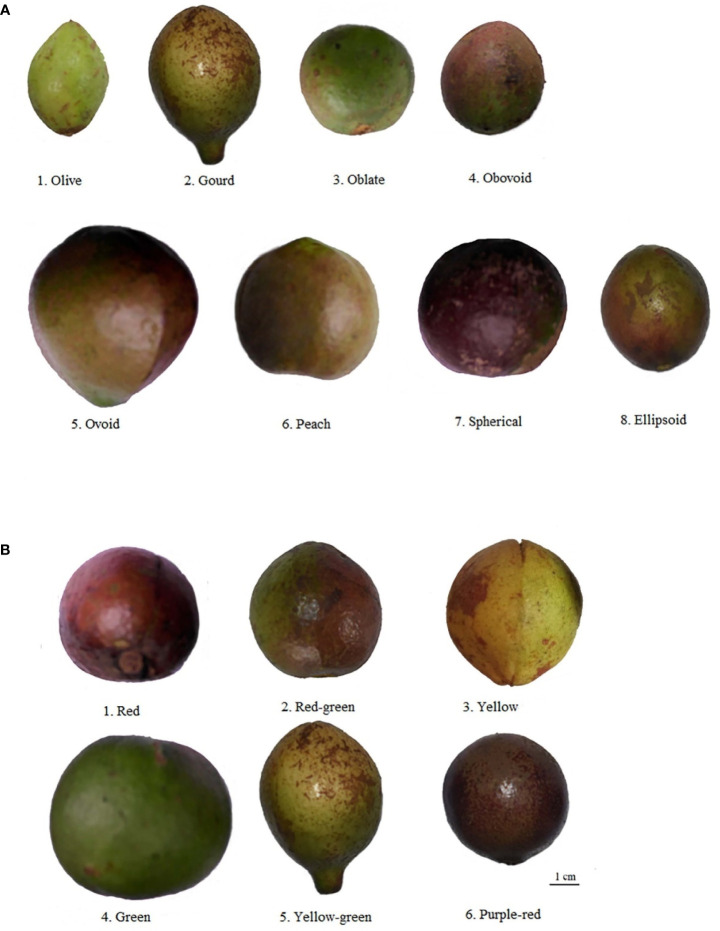
Rating of fruit morphologic characters for wild C. oleifera germplasm resources: **(A)** fruit shape and **(B)** peel color.

**Figure 5:**
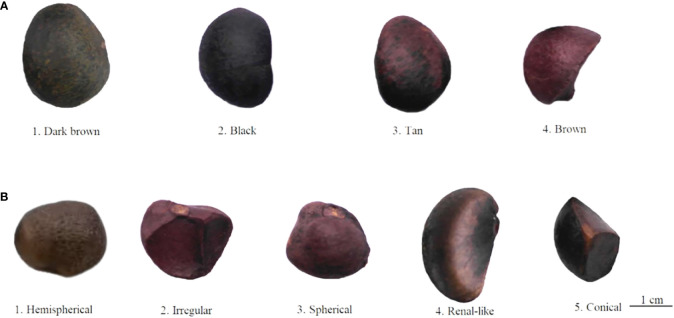
Rating of seed phenotypic traits for wild C. oleifera germplasm resources: **(A)** seed color and **(B)** seed shape.

H′, Shannon’s Diversity Index. Codes 1–9 correspond to the rating standard of leaf, flower, fruit, and seed characters, as shown in **Figures 2**–**5**.

**Table 2:** Variability and genetic diversity of the quantitative traits in leaves and flowers.

Traits	Max	Min	Mean	SD	Median	CV (%)	*H′*
Leaf traits							
Leaf length (mm)	81.94	50.21	67.02	7.48	67.07	11.16	2.06
Leaf width (mm)	44.70	19.99	32.49	5.31	33.27	16.36	2.07
Leaf area (mm^2^)	2,320.00	669.34	1,473.59	366.35	1,507.00	24.86	2.11
Leaf shape index	2.99	1.62	2.11	0.26	2.07	12.46	1.99
Flower traits							
Number of petals	9.38	5.00	7.24	0.86	7.00	11.81	1.78
Number of sepals	9.20	3.00	6.66	1.43	6.80	21.49	1.92
Number of stigmas	4.67	2.00	3.20	0.56	3.00	17.61	1.40
Flower crown area (mm^2^)	78.15	39.66	59.37	8.48	58.73	14.28	2.08
Petal length (mm)	36.91	19.41	28.93	3.54	29.04	14.28	2.06

The relative height between pistil and stamen was categorized into three categories among all 143 individuals: equal (36.36%), pistil higher (20.98%), and stamen higher (42.66%) (**Table 1**). The *H*′ for relative pistil and stamen height was 1.06 (**Table 1**). Additionally, white with crimson spots (1.4%) was observed in 143 wild *C. oleifera* resources (**Figure 3A**). The predominant petal color of 143 germplasm resources was white (98.60%), and very low phenotypic diversity was detected in petal color with an *H*′ value of 0.07 (**Table 1**). In addition, the stamen petalody, it should be noted, was a phenomenon that occurred with high frequency, representing the unstable flowering phenotype in wild *C.oleifera* resources (**Figure 3B**). Relatively high levels of diversity were also detected in flower quantitative traits; the CV for flower quantitative characteristics such as number of petals, number of sepals, number of stigmas, petal length, and flower crown ranged from 11.81% to 21.49%. The CV for the number of sepals was the greatest and ranged from 3.00 to 9.20. The *H*′ of flower quantitative traits varied between 1.40 and 2.08 (**Table 2**).

### 3.2 Fruit, seed phenotypic, and oil quality traits

**Figure 6:**
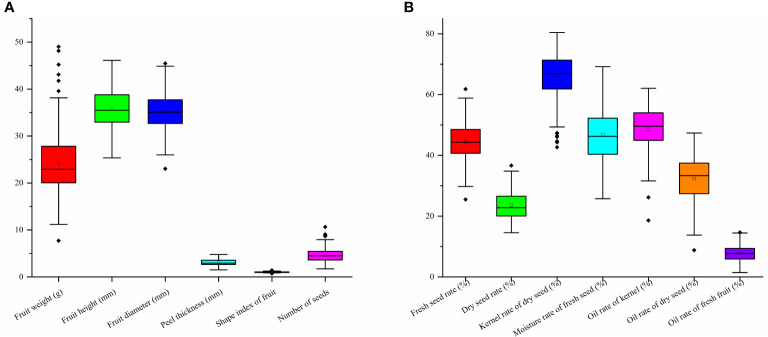
Fruit and oil content indexes variation of wild *C*. *oleifera* germplasm resources: **(A)** quantitative characters of fruit phenotypic characters and **(B)** quantitative characters of oil content indexes.

According to the morphologic variation in **Figure 4**, fruit shape was assigned eight ratings among the 143 accessions, the majority of which were spherical (52.45%), followed by peach (12.59%), oblate (10.49%), ovoid (9.09%), ellipsoid (8.39%), olive (4.90%), obovoid (1.40%), and gourd (0.70%) (**Figure 4A**; **Table 1**). The peel color was divided into six grades among the 143 individuals; the highest distribution frequency was green peel (47.55%), and the lowest was purple-red peel (0.70%). The red-green (22.38%) and yellow-green peel (24.48%) also covered a relatively high frequency (**Figure 4B**; **Table 1**). The *H*′ values for fruit shape and peel color were 1.50 and 1.59, respectively. The seed color was classified into four categories based on **Figure 5A**’s depiction; the types and distribution frequencies of each category were dark brown (23.08%), black (26.57%), tan (47.55%), and brown (2.8%), respectively. The seed shape was classified into five types, representing hemispherical (14.69%), irregular (20.98%), spherical (4.90%), renal-like (41.96%), and conical (17.48%), respectively (**Figure 5B**: **Table 1**). The *H*′ values of seed color and shape were 1.14 and 1.43, respectively (**Table 1**). The mean and median values of the fruit quantitative characteristics were consistent, representing the stability and typicality of the detected samples (**Figure 6**; **Supplementary Table S2**). The high level of CV values ranged from 10.22% to 35.05%, suggesting the richness of variability for the majority of these detected traits. Among these, the oil rate of fresh fruit had the largest CV value with a mean value of 7.79 and ranged from 1.45 to 14.64 (**Supplementary Table S2**). The shape index of fruit showed a lower level of diversity, with a CV value of 10.22% and a mean value of 1.02, ranging from 0.71 to 1.41. The results of fruit and economic characters presented similar levels of phenotypic diversity, with *H*′ values ranging from 1.96 to 2.07 (**Supplementary Table S2**).

Considering the analysis of fatty acid component content, the eicosenoic acid showed the highest variability (43.49%), ranging from 0.16% to 0.74% with a mean of 0.31%. As the main ingredient of fatty acid in *C. oleifera* oil, the oleic acid had the lowest variation (5.34%), with a range of 66.07%–83.71%, suggesting the relatively stable content of oleic acid in *C. oleifera* (**Table 3**). The *H*′ values of oil fatty acid characteristics ranged from 1.47 to 2.19, representing a higher level of diversity. For the oil quality index, the peroxide value had the highest CV of 81.31%, and the free acidity also holds a relatively high variation (41.96%). The *H*′ for free acidity and the peroxide value were 1.78 and 1.63, respectively (**Table 3**). The variability and genetic diversity were also detected in the lipid nutrient content. The *H*′ and CV values of α-tocopherol, squalene, and sterol were 1.90, 1.65, and 1.59 and 68.19%, 80.68%, and 72.66%, respectively (**Table 3**).

**Table 3:** Fatty acid component and oil quality characteristics for *C. oleifera*.

Traits	Max	Min	Median	Mean	SD	CV (%)	*H′*
Free acidity (mg/g)	1.47	0.19	0.40	0.43	0.18	41.96	1.78
Peroxide value (g/100g)	0.25	0.01	0.03	0.04	0.03	81.31	1.63
Palmitic acid (%)	14.07	8.35	11.51	11.47	0.98	8.54	2.19
Stearic acid (%)	4.69	1.04	1.99	2.12	0.56	26.20	1.77
Oleic acid (%)	83.71	66.07	77.33	77.16	4.12	5.34	1.90
Linoleic acid (%)	17.90	3.61	8.90	8.94	3.75	41.92	2.00
Eicosenoic acid (%)	0.74	0.16	0.29	0.31	0.14	43.49	1.47
Squalene (mg/kg)	273.03	18.57	69.02	92.40	63.01	68.19	1.90
α-Tocopherol (mg/kg)	630.07	4.05	169.19	168.04	135.58	80.68	1.65
Sterol (mg/kg)	9,910.00	656.37	1,876.61	3,297.07	2,395.69	72.66	1.59

### 3.3 Cluster analysis

**Figure 7:**
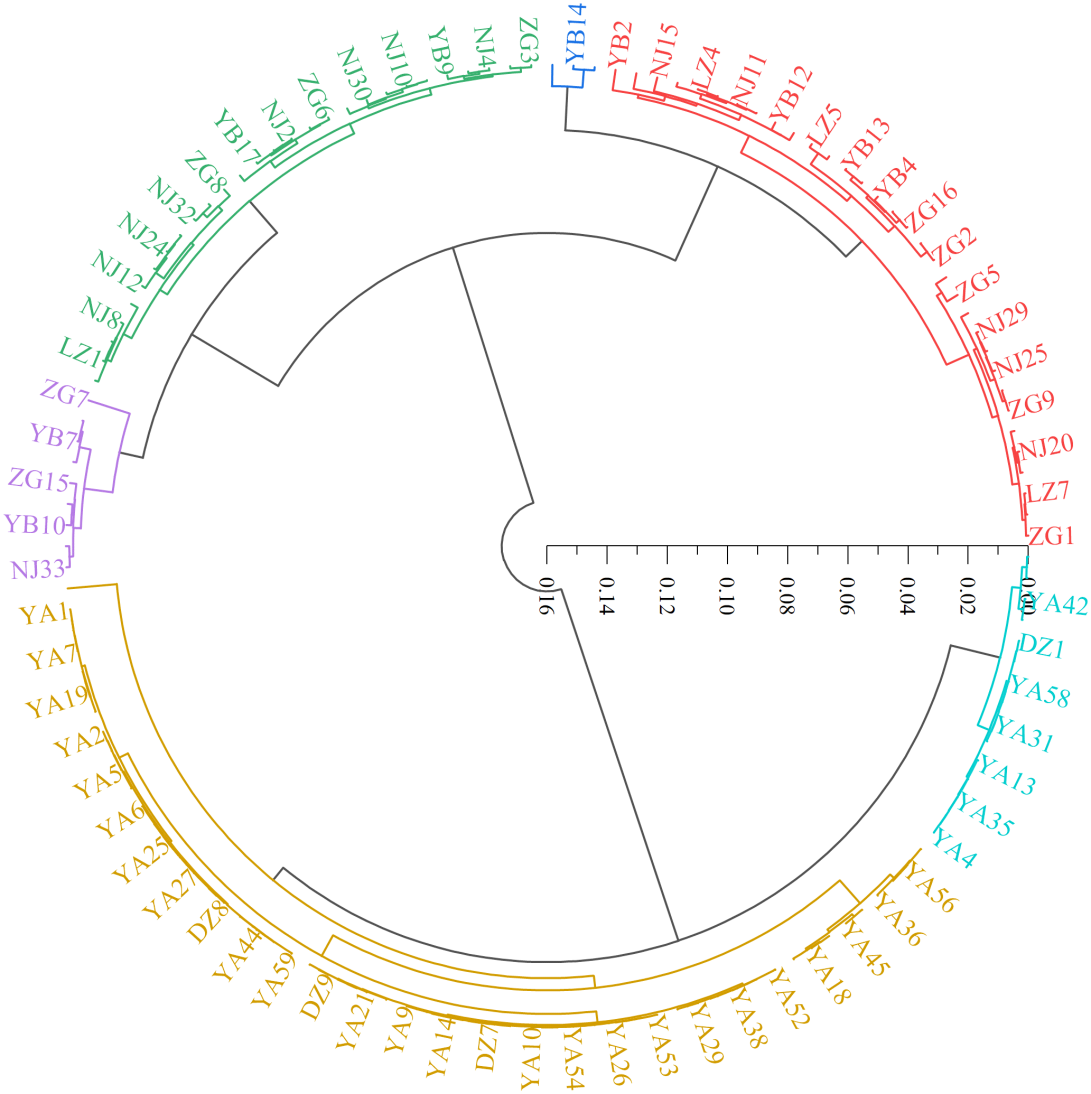
Cluster map of 143 C*. oleifera* germplasm resources.

In this study, hierarchical clustering was performed to analyze the relationship among the 143 wild *C. oleifera* germplasm samples. The result showed that all the accessions can be assigned to six distinct groups (**Figure 7**; **Supplementary Tables S3**). Group I contained 33 germplasm resources, representing 23.08% of the total accessions. This group was distinguished by the small leaves and high squalene content (**Figure 7**; **Supplementary Table S4**). Group II only consists of three germplasm resources (NJ1, YB14, and YB15) with small fruits, low oil content, high fresh seed rate, and tocopherol content. Group III contained 28 resources, which were separated by the high shape index of fruit and moderate other characteristics. Group IV contains nine individuals with large flowers and fruits, a high dry seed rate and oil rate of fresh fruit, and low free acidity and sterol content, all of which could be used as improved breeding materials. Group V contained 55 resources containing 38.46% of the total individuals, primarily from Ya’an. The characteristics of the group included high kernel rate of dry seed, oil rate of the kernel, oil yield, oleic acid and sterol content, low peroxide value, and moderate characteristics that could be used as improved breeding materials. Group VI contained 15 resources representing large leaves and fruits, high free acidity and oleic acid content and a small flower with low palmitic acid, squalene, and α-tocopherol content (**Figure 7**; **Supplementary Table S4**).

### 3.4 Correlation and principal component analyses

**Figure 8:**
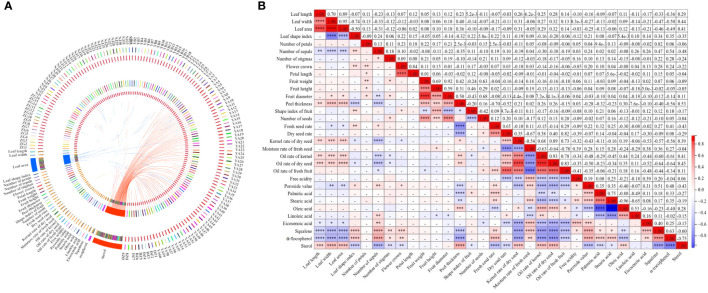
Correlation of 32 quantitative characteristics in 143 C*. oleifera* germplasm resources: **(A)** Circos visualization between traits and germplasm and **(B)** heat map for correlation analysis of–32 quantitative characteristics. Note: ^****^0.0001, ^***^0.001, and ^**^0.01—extremely significant correlation; ^*^0.05—significant correlation.

The Circos methodology was applied to better visualize and intuitively explore relational links among 32 quantitative characteristics with 143 wild *C. oleifera* germplasm resources (**Figure 8A**). Different abundances of 32 quantitative characteristics were detected in 143 accessions, among which the abundance of leaf area and sterol content was much higher than other traits. In addition, complex relationships among the 32 quantitative characteristics were estimated based on correlation analysis with the Pearson correlation coefficient in the 143 accessions (**Figure 8B**). Significant associations among leaves, flowers, fruits, seeds, and oil quality characteristics were shown, especially for some fruit traits. Strong positive correlations exist between fruit weight and fruit height, fruit diameter, peel thickness, and the number of seeds with the coefficient ranging from 0.42 to 0.92. Peel thickness had significantly negative correlations with fresh seed rate, dry seed rate, peroxide value, palmitic acid, stearic acid, squalene, and α-tocopherol contents, with the coefficient ranging from −0.23 to −0.70. Significant positive correlations also exist between oil content (oil rate of kernel, oil rate of dry seed, and oil rate of fresh fruit) and oleic acid (*r* = 0.18–0.44), and high and significant positive correlations were also shown among the three oil content indices. Strong negative correlations between oil rate of fresh fruit and other traits such as moisture rate of fresh seed, free acidity, peroxide value, stearic acid, eicosenoic acid, squalene, and α-tocopherol were tested, with coefficients ranging from −0.21 to −0.78. A positive correlation (*r* = 0.28) was also observed between oleic acid and sterol. Among all the 32 quantitative traits, the most positive correlation (*r* = 0.70) was detected between leaf length and leaf width, while the most negative (*r* = −0.96) was shown between the content of oleic acid and stearic acid (**Figure 8B**).

**Figure 9:**
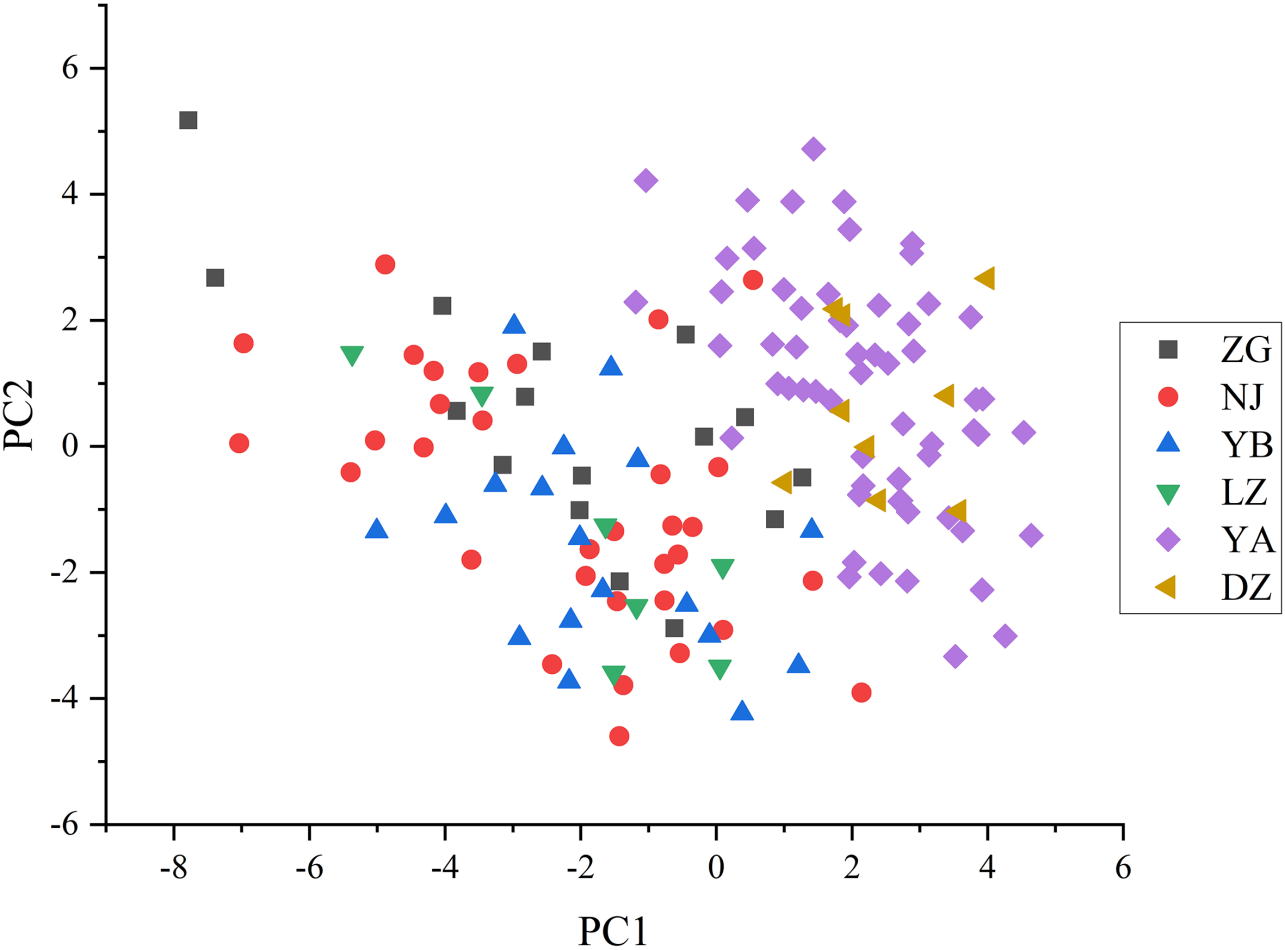
The principal component analysis of the 143 C*. oleifera* germplasm resources.

The PCA graph of 143 wild *C. oleifera* germplasm resources was obtained to demonstrate the distribution of accessions according to differences in quantitative phenotypic characteristics (**Figure 9**; **Supplementary Figure S1**). The results of PCA showed a distinctive separation among the 143 wild *C. oleifera* germplasm resources according to the phenotypic characteristics. Therefore, these phenotypic parameters can be used as an essential criterion for defining wild *C. oleifera* germplasm resources. Ulteriorly, The PCA was performed to identify the main distinguishing traits of the 32 quantitative characteristics. The dimension implied by the 32 quantitative characteristics was reduced to nine significant components, accounting for 80.79% of the total variance based on eigenvalues greater than 1 (**Figure S1**). The first factor, which accounted for 24.28% of the total variance comprised the kernel rate of dry seed, the oil rate of the kernel, and the kernel rate of dry seed (**Table 4**), so it can be referred to as the oil content factor. The fruit weight, diameter, and peel thickness had a higher loading on the second principal component, so it was referred to as the fruit yield factor. The third principal component, also known as the fatty acid factor, primarily represents fatty acids. The fourth component comprises the highest fruit weight load value. The petal length and flower crown had the highest load value, so the fifth factor was designated as the flower factor. In addition, the sixth, seventh, eighth, and ninth factors were referred to as leaf, fruit, free acidity, and fruit shape factors, respectively.

**Table 4:** The principal component analysis of the 32 quantitative characters in the 143 wild *C. oleifera* accessions.

Traits	Eigenvector of the principal component								
	1	2	3	4	5	6	7	8	9
Leaf length	0.45	0.28	0.11	−0.26	0.53	0.27	0.20	0.12	−0.25
Leaf width	0.61	0.43	0.12	−0.34	0.25	0.43	0.07	−0.07	0.11
Leaf area	0.59	0.41	0.12	−0.32	0.38	0.40	0.14	0.01	−0.04
Leaf shape index	−0.42	−0.34	−0.10	0.25	0.13	−0.36	0.11	0.22	−0.39
Number of petals	−0.07	0.20	0.17	0.11	0.38	0.16	−0.07	−0.54	0.15
Number of sepals	−0.48	−0.29	−0.16	0.16	0.06	0.33	0.08	−0.18	0.30
Number of stigmas	−0.26	0.09	0.26	0.24	−0.12	0.21	−0.32	0.36	0.28
Flower crown	−0.27	−0.01	0.02	0.15	0.77	−0.23	−0.39	−0.03	−0.03
Petal length	−0.10	0.00	−0.04	0.05	0.74	−0.26	−0.52	0.05	−0.05
Fruit weight	−0.06	0.63	0.31	0.65	0.05	−0.01	0.14	0.00	0.01
Fruit height	−0.06	0.50	0.24	0.33	0.28	−0.37	0.53	0.12	0.22
Fruit diameter	0.14	0.63	0.35	0.64	−0.04	0.03	0.05	−0.01	−0.10
Peel thickness	0.54	0.61	−0.21	0.22	−0.08	−0.19	−0.09	0.04	0.16
Shape index of fruit	−0.23	−0.13	−0.12	−0.32	0.36	−0.46	0.51	0.17	0.37
Number of seeds	0.05	0.20	0.56	0.61	−0.20	−0.01	−0.09	−0.10	−0.10
Fresh seed rate	−0.38	−0.39	0.50	−0.02	0.11	0.21	0.34	−0.02	−0.33
Dry seed rate	0.10	−0.72	0.57	0.15	0.14	0.17	0.14	0.04	0.01
Kernel rate of dry seed	0.74	−0.26	0.33	−0.08	−0.06	−0.18	−0.04	0.04	0.04
Moisture rate of fresh seed	−0.49	0.56	−0.27	−0.22	−0.04	−0.05	0.17	−0.07	−0.33
Oil rate of kernel	0.82	−0.36	0.14	0.13	0.05	−0.07	−0.05	0.04	0.01
Oil rate of dry seed	0.85	−0.34	0.25	0.04	0.00	−0.13	−0.06	0.04	0.02
Oil rate of fresh fruit	0.57	−0.62	0.49	0.11	0.09	0.01	0.03	0.05	0.02
Free acidity	−0.27	0.33	0.08	−0.08	−0.05	0.29	−0.12	0.60	0.15
Peroxide value	−0.76	−0.09	0.10	0.07	0.11	0.08	0.00	−0.04	0.05
Palmitic acid	−0.43	0.07	0.59	−0.44	−0.07	−0.25	−0.06	0.05	0.08
Stearic acid	−0.47	0.28	0.59	−0.44	−0.13	−0.06	−0.15	−0.07	0.05
Oleic acid	0.53	−0.19	−0.61	0.44	0.10	0.09	0.13	0.02	−0.08
Linoleic acid	0.15	−0.39	−0.47	0.38	0.15	0.25	0.08	0.03	0.21
Eicosenoic acid	−0.30	−0.07	−0.05	0.10	0.17	0.33	−0.02	0.41	−0.19
Squalene	−0.78	−0.07	−0.17	0.14	0.09	0.12	−0.04	0.05	0.00
α-Tocopherol	−0.84	−0.16	0.07	0.10	0.02	0.13	0.02	−0.19	−0.01
Sterol	0.70	0.29	−0.16	−0.21	−0.13	−0.17	−0.14	0.02	−0.17

### 3.5 M-TOPSIS comprehensive evaluation

The TOPSIS method incorporating the Mahalanobis distance (M-TOPSIS) is used for comprehensively evaluating germplasm. It is a novel, modified, and more practical synthetic evaluation method than TOPSIS. Here, 11 indexes including leaf width, number of sepals, flower crown, fruit weight, fruit height, fruit diameter, shape index of fruit, oil rate of dry seed, free acidity, stearic acid, and eicosenoic acid contents were selected from 32 quantitative traits to evaluate the 143 wild *C. oleifera* germplasm resources in all aspects based on correlation and PCA analyses. Following that, a comprehensive evaluation model of *C. oleifera* resources was constructed by the M-TOPSIS, and the top 10 accessions were screened as YA53, YA13, YA40, YA34, YA57, YA19, YA33, YA41, DZ8, and YA7 (**Table 5**).

**Table 5:** Comprehensive score and ranking of 143 C*. oleifera* germplasm resources.

Code	Comprehensive score	Rank	Code	Comprehensive score	Rank	Code	Comprehensive score	Rank
ZG1	0.313	93	YB1	0.331	84	YA25	0.493	22
ZG2	0.339	81	YB2	0.278	106	YA26	0.493	22
ZG3	0.388	58	YB3	0.329	85	YA27	0.354	73
ZG4	0.279	105	YB4	0.287	102	YA28	0.467	26
ZG5	0.316	90	YB5	0.294	99	YA29	0.396	56
ZG6	0.441	37	YB6	0.314	92	YA30	0.443	35
ZG7	0.396	56	YB7	0.349	74	YA31	0.431	40
ZG8	0.294	99	YB8	0.297	97	YA32	0.435	39
ZG9	0.367	68	YB9	0.322	87	YA33	0.568	7
ZG10	0.276	107	YB10	0.329	85	YA34	0.583	4
ZG11	0.325	86	YB11	0.342	79	YA35	0.437	38
ZG12	0.348	75	YB12	0.382	61	YA36	0.392	57
ZG13	0.423	45	YB13	0.410	50	YA37	0.518	18
ZG14	0.418	46	YB14	0.252	108	YA38	0.446	34
ZG15	0.452	31	YB15	0.295	98	YA39	0.398	54
ZG16	0.356	71	YB16	0.388	58	YA40	0.599	3
NJ1	0.354	73	YB17	0.344	78	YA41	0.563	8
NJ2	0.447	33	YB18	0.378	65	YA42	0.548	12
NJ3	0.378	65	LZ1	0.313	93	YA43	0.550	11
NJ4	0.454	43	LZ2	0.340	80	YA44	0.403	52
NJ5	0.392	103	LZ3	0.319	88	YA45	0.427	42
NJ6	0.310	101	LZ4	0.283	104	YA46	0.411	49
NJ7	0.451	95	LZ5	0.334	82	YA47	0.381	62
NJ8	0.411	49	LZ6	0.412	48	YA48	0.380	63
NJ9	0.381	62	LZ7	0.322	87	YA49	0.381	62
NJ10	0.334	82	YA1	0.334	82	YA50	0.409	51
NJ11	0.322	87	YA2	0.534	14	YA51	0.428	41
NJ12	0.344	79	YA3	0.383	60	YA52	0.317	89
NJ13	0.428	41	YA4	0.476	24	YA53	0.623	1
NJ14	0.530	16	YA5	0.427	42	YA54	0.533	15
NJ15	0.332	83	YA6	0.356	71	YA55	0.399	53
NJ16	0.319	88	YA7	0.552	10	YA56	0.369	67
NJ17	0.360	69	YA8	0.458	28	YA57	0.580	5
NJ18	0.293	100	YA9	0.467	26	YA58	0.529	17
NJ19	0.315	91	YA10	0.517	19	YA59	0.545	13
NJ20	0.357	70	YA11	0.423	45	YA60	0.424	44
NJ21	0.373	66	YA12	0.485	23	DZ1	0.392	57
NJ22	0.357	70	YA13	0.601	2	DZ2	0.397	55
NJ23	0.162	109	YA14	0.501	20	DZ3	0.459	27
NJ24	0.154	110	YA15	0.498	21	DZ4	0.355	72
NJ25	0.345	77	YA16	0.442	36	DZ5	0.379	64
NJ26	0.283	104	YA17	0.471	25	DZ6	0.355	72
NJ27	0.346	76	YA18	0.385	59	DZ7	0.435	39
NJ28	0.455	29	YA19	0.576	6	DZ8	0.562	9
NJ29	0.334	82	YA20	0.441	37	DZ9	0.299	96
NJ30	0.426	43	YA21	0.442	36			
NJ31	0.286	103	YA22	0.424	44			
NJ32	0.292	101	YA23	0.355	72			
NJ33	0.300	95	YA24	0.415	47			

## 4 Discussion

### 4.1 Phenotypic variations of wild *C. oleifera* germplasm

The coefficient of variation can reflect the degree of difference between various phenotypic traits. A strong positive correlation has been reported between the coefficient of variation and the degree of phenotypic difference as well as genetic diversity. It provided a stronger possibility for using phenotypic traits to identify varieties and germplasms (Zhang et al., 2022). Based on the analysis of 41 phenotypic traits of 143 germplasms, significant phenotypic differences were found among various wild *C. oleifera* germplasms. During the 2 years of investigation, the high degree of phenotypic variation indicated the abundant genetic diversity existing in *C. oleifera* germplasms. Some workers have also reported similar results in *C. oleifera* and its relatives, *C. meiocarpa* (Huang, 2011; He et al., 2020). In the study, the median and mean values of quantitative characteristics from 143 germplasms were nearly identical, indicating that the investigated germplasm resources were representative. The higher value of *H*′ (1.15–2.11) was detected in leaf traits, reflecting the greater genetic variation in these characters (**Tables 1**, **2**). The leaf trait has been considered an important index in plant science research for it reflects the adaptability of plants to different environments and their ability to self-regulate in response to complex physiological environments (Hu et al., 2022).

Flower variation is the raw material that natural selection can amplify, resulting in plant diversification over time (Herrera, 2005). A significant difference was shown in flower phenotypic traits with *H*′ from 0.07 to 2.08, implying the foundation of these traits for variety breeding and excellent germplasm choosing (**Tables 1**, **2**). In addition, the stamen petalody was a surprising phenomenon during our investigation (**Figure 3B**). The stamen of the male sterile mutant shows remarkable petalody, increasing whorls of petals and generating different flower forms, which are extremely important traits for ornamental value and serve as a useful genetic tool because they eliminates the need for artificial emasculation (Li et al., 2021).

The primary reason for cultivating *C. oleifera* is to extract edible oil. The seeds of *C. oleifera* are the primary oil storage components. The characteristics of *C. oleifera* seeds are crucial for oil yield and quality and are essential for DUS testing of Oil-tea *Camellia* (Zhu et al., 2020). Moreover, fruit traits are the essential phenotypic traits for fruit-producing economic tree species, which directly or indirectly affect the yield of *C.oleifera* (Zhu et al., 2020). The fruit phenotype is crucial for further elucidating genetic diversity and developing superior varieties, and the fruit descriptors system of *C. oleifera* should be supplemented and completed with more fruit characteristics, such as the fruit shape should be added with peach, olive, and gourd (**Figure 4**). In this study, the CV for oil content of fresh fruit (35.05%) from 143 wild *C. oleifera* resources was the highest and the degree of data dispersion was the greatest (**Supplementary Table S2**). As an important woody oil crop, the oil content of fresh fruit is directly proportional to oil production and economic value (Hao et al., 2017). Research on fruit traits is critical and closely related to oil yield, an important breeding and variety-promotion reference (Yang et al., 2022).

The oil extracted from *C. oleifera* is a kind of high-quality edible oil rich in many bioactive substances (Zhu et al., 2019). In this study, the CV values of peroxide value, α-tocopherol, sterol, and squalene were 81.31%, 80.68%, 72.66%, and 68.19%, respectively (**Table 3**). It indicated that these four traits have an enormous genetic variation (Kumar et al., 2020). On the contrary, the low level of CV for palmitic acid and oleic acid was detected here, which indicated that the variation was slight and the genetic characteristics were stable.

### 4.2 Cluster analysis of phenotypic traits in *C. oleifera* germplasm resources

The cluster analysis can gather varieties with similar genetic information into one group, which is conducive to studying the genetic relationship between varieties (Dalmaijer et al., 2022). In this study, the wildest *C. oleifera* germplasms from YA were clustered into one group (**Figure 9**), indicating the existence of a certain correlation between phenotypic traits and geographical location among different materials. However, the geographical origin might not play a decisive role in phenotypic traits, some of the accessions from different geographical locations in this study were clustered together (**Figure 9**). Genetic variation may be occurring frequently when materials from various sources adopted significant habitat differences, which may be caused by the differences between the original environment and the present habitat (Singh et al., 2013). Except for the differences in genetic material, it is also possible that the introduction of germplasm materials to the local long-term planting caused differences in their growth and development (Kumar et al., 2009). In addition, there was obvious phenotypic differentiation among different groups, which can provide optimized germplasm materials for varieties breeding of *C.oleifera.* The richness of various traits not only improves the efficiency of breeding and speeds up the process of breeding but also provides a fundamental reference for the selection of hybrid parents and the optimization of combinations in breeding (You, 2021).

### 4.3 Correlation and principal component analyses between the quantitative traits

This study observed significant negative correlations between peel thickness and fresh seed rate, dry seed rate, peroxide value, palmitic acid, squalene, and α-tocopherol (**Figure 8**), which was consistent with previous findings (He et al., 2020). There were significant positive correlations between oil content and oleic acid, linoleic acid, and sterol, indicating the possibility of excellent resource selection with high oil yield and nutrition content. Significant negative correlations were observed between oleic acid, palmitic acid, and stearic acid. The result agreed with the previous study that unsaturated fatty acid enzymes predominantly catalyze the formation of fatty acids and that oleic acids are formed after the prolongation and desaturation of palmitic acid and stearic acid during the synthesis of fatty acids in plants (Zhao et al., 2015).

PCA is an effective method for reducing the dimensionality of large datasets, which can maximize interpretability, minimize information loss, and determine the most suitable traits that mostly contribute to the variation in the selected materials (Nardo et al., 2005; Jolliffe and Cadima, 2016). In this study, PCA confirmed that the first nine components explained the vast majority of the variation, concentrating on several characteristics, such as the oil content of dry seed, fruit diameter, stearic acid, fruit weight, petal length, leaf width, fruit height, free acidity, and fruit shape indexes (**Figure 9**; **Figure S1**). The results suggested that such characters are suitable both for the assessment of genetic diversity and for the phenotypic characterization of wild *C. oleifera* germplasm.

## 5 Conclusions

In this study, 41 phenotypic characteristics (one qualitative, eight pseudo-qualitative, and thirty-two quantitative) including leaves, flowers, fruits, seeds, and oil quality traits were observed to assess the diversity of 143 wild *C. oleifera* germplasm resources. Ample phenotypic variations were exhibited in the accessions. Meanwhile, the DUS index system for *C. oleifera* was supplemented and reestablished with statistical phenotypic characters. The results of this study will contribute to expanding the descriptor system and optimizing the DUS test guideline of oil-tea *Camellia*. In addition, it will provide a reference for further utilization of *C. oleifera* germplasm resources and genetic improvement of main characters, and consolidate the theoretical basis for breeding new varieties of *C. oleifera* in the future.

## Data availability statement

The original contributions presented in the study are included in the article/**Supplementary Material**. Further inquiries can be directed to the corresponding author.

## Author contributions

TC: investigation, formal analysis, methodology, and writing—review and editing. LL: investigation, methodology, and writing—original draft. YZ: methodology and investigation. QZ: methodology and investigation. SL: investigation and software. TX: investigation and software. LZ: supervision and software. SF: methodology. HY: supervision. CD: supervision, conceptualization, and funding acquisition. All authors contributed to the article and approved the submitted version.
