# Supplementary material for: Characterization and comprehensive evaluation of phenotypic characters in wild Camellia oleifera germplasm for conservation and breeding
Source: Front Plant Sci. 2023 Mar 21;14:1052890. doi: 10.3389/fpls.2023.1052890 (PMC10070972; doi:10.3389/fpls.2023.1052890)

Supplementary Material

**TABLE S1.** The list of qualitative and pseudo-qualitative characters and their measures.

| Traits | Trait number | Trait characteristics |
| --- | --- | --- |
| Qualitative characters |  |  |
| Relative height of Pistil and stamen | 3 | Equal, pistil higher and stamen higher |
| pseudo-qualitative characters |  |  |
| Leaf shape | 5 | Subcircular, oval, lanceolate, long elliptical and elliptical |
| Shape of leaf apex | 4 | Taper, blunt, round, and sharp |
| Petal color | 2 | White, white with crimson spots |
| Stamen development | 2 | Normal and petalization |
| Fruit shape | 8 | Olive, gourd, oblate, obovoid, ovoid, peach, spherical, and ellipsoid |
| Peel color | 6 | Red, red-green, yellow, green, yellow-green,  and purple-red |
| Seed color | 4 | Dark brown, black, tan and brown |
| Seed shape | 5 | Hemispherical, irregular, spherical, renal-like and conical |

**TABLE S2.** Fruit quantitative traits of 143 wild *C. oleifera* germplasm resources**.**

| Accessions | | Fruit weight (g) | Fruit height (mm) | Fruit diameter (mm) | Peel thickness (mm) | Shape index of fruit | Number of seeds | Fresh seed rate (%) | Dry seed rate (%) | Kernel rate of dry seed (%) | Moisture rate of fresh seed (%) | Oil rate of kernel (%) | Oil rate of dry seed (%) | Oil rate of fresh fruit (%) |
| --- | --- | --- | --- | --- | --- | --- | --- | --- | --- | --- | --- | --- | --- | --- |
| ZG1 | 27.81±5.44 | 35.96±2.93 | 36.12±3.76 | 2.67±0.32 | 1.00±0.11 | 4.93±1.63 | 48.35±0.64 | 26.25±0.16 | 58.52±3.16 | 45.70±4.16 | 50.90±1.30 | 29.78±0.44 | 7.82±0.51 |  |
| ZG2 | 27.24±4.26 | 35.31±2.08 | 37.01±2.59 | 3.23±0.43 | 0.96±0.08 | 4.90±1.51 | 43.10±1.34 | 20.26±0.48 | 54.57±4.54 | 53.01±1.22 | 38.77±1.46 | 21.15±1.34 | 4.29±0.08 |  |
| ZG3 | 38.13±4.94 | 40.57±1.83 | 40.78±2.46 | 3.44±0.37 | 0.99±0.05 | 6.40±1.33 | 49.00±2.34 | 24.11±1.09 | 56.20±3.48 | 50.80±2.16 | 47.00±0.99 | 26.42±0.46 | 6.37±0.64 |  |
| ZG4 | 22.20±2.57 | 35.87±2.24 | 34.54±1.59 | 2.61±0.37 | 1.03±0.07 | 4.73±1.00 | 46.50±0.62 | 22.17±2.01 | 55.59±1.49 | 52.33±2.44 | 42.10±1.67 | 23.41±0.88 | 5.19±0.16 |  |
| ZG5 | 20.76±2.53 | 30.81±1.36 | 34.96±5.72 | 2.83±0.36 | 0.89±0.09 | 7.70±2.05 | 46.27±0.34 | 29.91±1.46 | 68.08±5.01 | 35.31±1.80 | 48.53±2.66 | 33.03±1.00 | 9.87±0.91 |  |
| ZG6 | 34.25±4.04 | 39.62±2.38 | 39.68±4.04 | 3.23±0.40 | 1.00±0.11 | 4.50±1.09 | 47.17±2.31 | 21.23±1.11 | 64.23±4.21 | 55.00±2.65 | 46.46±2.48 | 29.82±0.64 | 6.33±0.50 |  |
| ZG7 | 27.02±6.06 | 34.07±2.49 | 37.30±4.50 | 2.72±0.38 | 0.92±0.08 | 4.76±1.67 | 44.52±3.64 | 22.03±1.18 | 55.78±3.16 | 50.42±3.44 | 48.76±0.88 | 27.20±0.17 | 6.00±0.09 |  |
| ZG8 | 24.35±3.35 | 36.47±1.98 | 34.75±2.13 | 3.18±0.46 | 1.05±0.07 | 3.30±1.24 | 43.41±3.09 | 20.39±2.06 | 57.53±2.49 | 52.65±2.00 | 42.10±0.46 | 24.23±1.07 | 4.99±0.29 |  |
| ZG9 | 28.32±3.32 | 36.02±2.55 | 36.94±2.70 | 3.40±0.36 | 0.98±0.10 | 4.53±1.02 | 43.22±3.07 | 22.81±2.18 | 67.15±5.00 | 47.15±1.99 | 42.68±2.01 | 28.66±0.62 | 6.53±0.11 |  |
| ZG10 | 26.83±4.72 | 35.85±1.99 | 35.19±4.06 | 2.46±0.39 | 1.06±0.23 | 4.83±1.55 | 47.85±2.61 | 17.02±0.79 | 44.67±4.16 | 64.52±3.80 | 34.59±1.44 | 15.45±0.08 | 2.64±0.01 |  |
| ZG11 | 23.99±5.17 | 40.99±3.33 | 32.84±3.59 | 2.81±0.32 | 1.25±0.13 | 3.26±1.12 | 45.26±1.64 | 19.41±0.88 | 52.56±2.05 | 56.98±2.79 | 26.17±0.99 | 13.75±0.04 | 2.67±0.01 |  |
| ZG12 | 39.58±7.80 | 41.11±2.26 | 40.95±4.08 | 2.74±0.23 | 1.01±0.08 | 6.11±1.89 | 53.49±1.69 | 16.47±1.06 | 47.38±3.55 | 69.19±3.47 | 18.58±0.48 | 8.81±0.10 | 1.45±0.02 |  |
| ZG13 | 24.71±3.28 | 43.58±3.66 | 33.69±2.82 | 2.90±0.41 | 1.3±0.15 | 4.46±1.33 | 46.69±0.99 | 27.85±1.27 | 66.95±3.46 | 40.38±4.62 | 51.92±1.60 | 34.75±0.37 | 9.69±0.16 |  |
| ZG14 | 25.80±3.74 | 33.67±2.82 | 35.09±3.34 | 2.48±0.26 | 0.96±0.07 | 3.20±0.79 | 54.68±3.07 | 30.88±1.49 | 67.97±3.71 | 43.54±3.00 | 50.22±1.08 | 34.14±0.67 | 10.54±0.15 |  |
| ZG15 | 31.97±3.49 | 36.80±4.05 | 38.05±3.17 | 2.88±0.39 | 0.97±0.13 | 5.13±1.12 | 46.55±2.16 | 23.11±0.99 | 64.43±2.91 | 50.37±3.99 | 49.18±2.00 | 31.69±0.55 | 7.32±0.60 |  |
| ZG16 | 21.04±3.38 | 46.10±2.01 | 32.79±2.26 | 2.75±0.30 | 1.41±0.06 | 4.20±1.32 | 40.18±0.67 | 23.74±0.84 | 57.83±2.18 | 40.91±2.78 | 51.10±1.67 | 29.55±0.71 | 7.01±0.10 |  |
| NJ1 | 24.79±4.26 | 35.40±2.11 | 35.27±2.14 | 2.36±0.44 | 1.00±0.07 | 5.83±1.98 | 56.17±0.98 | 23.39±2.12 | 56.65±4.66 | 58.38±2.44 | 38.24±0.48 | 21.67±0.09 | 5.07±0.52 |  |
| NJ2 | 28.65±5.12 | 37.32±2.19 | 37.73±3.00 | 2.06±0.20 | 0.99±0.06 | 6.60±2.08 | 57.87±2.61 | 36.66±1.44 | 69.03±2.78 | 36.65±1.66 | 54.99±1.66 | 37.96±0.77 | 13.92±1.00 |  |
| NJ3 | 33.90±6.71 | 41.49±3.41 | 37.70±5.19 | 2.85±0.31 | 1.12±0.21 | 4.53±1.38 | 48.24±3.07 | 25.97±1.64 | 55.92±3.77 | 46.16±3.44 | 46.32±0.71 | 25.90±1.04 | 6.73±0.61 |  |
| NJ4 | 34.54±5.61 | 40.72±2.20 | 40.21±2.94 | 2.89±0.33 | 1.01±0.07 | 7.63±2.02 | 25.49±0.62 | 16.52±1.58 | 64.77±2.06 | 35.23±2.15 | 45.24±0.64 | 29.32±0.61 | 4.86±0.49 |  |
| NJ5 | 45.21±7.43 | 43.90±2.58 | 43.01±3.41 | 2.91±0.33 | 1.02±0.08 | 7.93±2.03 | 49.89±1.94 | 19.88±1.78 | 44.29±2.46 | 60.14±2.88 | 34.45±1.06 | 15.27±0.82 | 3.04±0.06 |  |
| NJ6 | 23.53±3.67 | 35.96±2.00 | 34.11±2.41 | 2.83±0.37 | 1.05±0.08 | 3.93±1.00 | 46.73±2.61 | 25.32±1.64 | 65.60±2.22 | 45.69±1.80 | 48.18±0.88 | 31.60±1.11 | 8.00±0.30 |  |
| NJ7 | 20.38±2.66 | 33.15±1.70 | 33.51±2.07 | 2.66±0.23 | 0.99±0.06 | 5.83±1.49 | 46.24±1.79 | 31.52±0.81 | 74.51±2.19 | 31.84±1.66 | 56.37±1.60 | 42.01±0.66 | 13.25±0.27 |  |
| NJ8 | 26.29±5.03 | 32.47±1.87 | 37.70±2.69 | 2.62±0.25 | 0.86±0.06 | 7.53±2.38 | 48.95±1.64 | 29.09±0.77 | 63.45±3.44 | 40.62±2.57 | 50.85±2.11 | 32.29±0.83 | 9.43±0.60 |  |
| NJ9 | 25.09±4.92 | 40.05±3.13 | 35.04±3.34 | 2.58±0.25 | 1.14±0.07 | 4.66±1.35 | 50.77±1.66 | 26.56±1.02 | 60.55±1.46 | 47.69±3.00 | 47.25±0.55 | 28.59±1.34 | 7.60±0.49 |  |
| NJ10 | 25.92±3.02 | 37.74±2.40 | 36.92±2.16 | 2.79±0.35 | 1.02±0.06 | 6.90±1.42 | 52.64±2.49 | 28.70±1.00 | 67.78±2.71 | 45.50±2.54 | 49.19±1.04 | 33.34±0.57 | 9.58±0.27 |  |
| NJ11 | 35.35±5.43 | 40.81±2.73 | 40.07±2.78 | 2.90±0.26 | 1.02±0.08 | 5.10±1.04 | 51.17±2.67 | 25.24±2.49 | 59.70±1.88 | 50.68±2.89 | 43.66±1.44 | 26.06±0.70 | 6.58±0.08 |  |
| NJ12 | 29.46±2.55 | 34.64±1.28 | 38.82±1.71 | 2.93±0.22 | 0.89±0.04 | 6.50±1.59 | 49.81±2.16 | 22.45±0.67 | 58.22±1.69 | 54.94±3.67 | 39.01±1.08 | 22.71±0.61 | 5.10±0.07 |  |
| NJ13 | 34.77±4.38 | 41.42±2.11 | 40.45±4.38 | 2.79±0.21 | 1.03±0.10 | 6.33±1.51 | 57.18±0.64 | 34.83±0.55 | 70.22±2.49 | 39.10±2.01 | 53.95±1.30 | 37.88±0.44 | 13.20±0.60 |  |
| NJ14 | 49.03±6.68 | 43.16±2.04 | 45.46±2.60 | 4.21±0.44 | 0.95±0.04 | 8.66±1.68 | 44.65±0.67 | 24.09±1.49 | 64.54±3.01 | 45.99±1.54 | 44.75±1.85 | 28.90±0.82 | 6.96±0.18 |  |
| NJ15 | 43.09±4.87 | 32.35±4.10 | 44.87±4.24 | 3.03±0.29 | 0.71±0.06 | 9.06±1.67 | 29.75±1.20 | 19.25±1.17 | 58.95±2.09 | 33.53±2.00 | 33.73±0.79 | 19.88±0.67 | 3.83±0.05 |  |
| NJ16 | 13.75±1.95 | 32.94±2.06 | 27.02±1.92 | 1.78±0.21 | 1.22±0.06 | 2.56±0.67 | 55.47±2.94 | 33.48±1.48 | 68.51±2.11 | 39.64±1.01 | 40.05±0.55 | 27.40±0.32 | 9.17±0.11 |  |
| NJ17 | 24.00±4.81 | 36.46±1.69 | 34.74±1.44 | 2.33±0.33 | 1.05±0.06 | 6.40±1.54 | 57.32±2.64 | 30.49±1.55 | 72.16±1.64 | 46.77±1.49 | 48.67±1.07 | 35.12±0.22 | 10.71±0.42 |  |
| NJ18 | 22.05±3.48 | 37.42±3.21 | 32.41±2.62 | 2.49±0.50 | 1.15±0.12 | 2.60±0.66 | 46.97±0.61 | 23.99±1.61 | 52.97±0.99 | 48.92±1.77 | 37.48±0.55 | 19.83±0.81 | 4.76±0.80 |  |
| NJ19 | 21.00±2.60 | 33.75±3.28 | 34.19±1.92 | 1.50±0.44 | 0.98±0.09 | 5.16±1.19 | 61.80±3.09 | 26.19±1.67 | 58.80±1.64 | 57.62±2.80 | 32.08±0.67 | 18.87±1.01 | 4.94±0.06 |  |
| NJ20 | 17.14±2.10 | 33.90±2.68 | 30.15±2.56 | 2.03±0.91 | 1.13±0.12 | 2.33±0.62 | 50.67±0.46 | 26.41±1.88 | 66.85±2.68 | 47.92±3.01 | 33.83±1.49 | 22.60±0.19 | 5.95±0.05 |  |
| NJ21 | 25.50±3.73 | 37.45±2.32 | 36.65±2.13 | 2.10±0.74 | 1.02±0.08 | 5.60±1.20 | 58.86±1.67 | 32.98±0.94 | 62.81±0.87 | 43.71±3.00 | 51.75±2.00 | 32.50±0.90 | 10.71±0.16 |  |
| NJ22 | 33.52±4.14 | 44.49±3.15 | 38.80±2.45 | 2.58±0.53 | 1.15±0.10 | 7.10±1.60 | 52.32±2.71 | 33.35±2.18 | 71.45±1.66 | 36.27±0.59 | 57.24±1.66 | 40.90±1.61 | 13.65±0.22 |  |
| NJ23 | 12.64±2.76 | 32.82±1.74 | 25.98±2.34 | 2.26±0.36 | 1.26±0.08 | 2.06±1.93 | 42.01±1.67 | 16.53±2.10 | 47.23±1.85 | 60.61±1.86 | 31.60±1.07 | 14.91±0.11 | 2.47±0.01 |  |
| NJ24 | 14.34±2.96 | 29.73±2.07 | 27.76±2.57 | 2.58±0.32 | 1.07±0.09 | 2.50±0.67 | 42.05±2.55 | 17.34±1.00 | 46.39±2.67 | 58.73±4.01 | 37.42±1.00 | 17.40±0.59 | 3.01±0.31 |  |
| NJ25 | 20.31±2.43 | 37.22±1.52 | 31.91±2.23 | 2.40±0.37 | 1.17±0.09 | 4.33±1.16 | 55.54±0.64 | 33.20±0.46 | 68.81±3.18 | 40.22±2.19 | 54.44±1.44 | 37.46±1.00 | 12.44±0.08 |  |
| NJ26 | 17.38±3.12 | 34.23±2.70 | 30.03±2.82 | 2.15±0.33 | 1.14±0.14 | 4.83±1.27 | 54.34±0.82 | 33.18±1.84 | 66.78±3.10 | 38.87±2.00 | 53.23±0.55 | 35.55±0.71 | 11.80±0.64 |  |
| NJ27 | 24.05±3.17 | 35.47±2.39 | 35.16±2.03 | 2.98±0.35 | 1.01±0.08 | 4.33±1.40 | 46.83±1.49 | 25.36±1.44 | 68.09±2.66 | 45.93±1.66 | 52.46±0.26 | 35.72±0.61 | 9.08±0.44 |  |
| NJ28 | 29.17±3.20 | 39.19±1.57 | 37.10±2.33 | 2.85±0.34 | 1.05±0.05 | 5.83±1.39 | 50.64±1.77 | 28.64±1.73 | 71.76±3.18 | 43.45±1.80 | 49.33±0.41 | 35.40±0.50 | 10.14±0.34 |  |
| NJ29 | 22.94±3.18 | 32.80±1.68 | 33.96±2.71 | 2.66±0.54 | 0.97±0.08 | 5.23±1.48 | 46.68±0.88 | 22.87±1.72 | 68.46±2.37 | 51.01±3.17 | 50.28±2.16 | 34.42±0.92 | 7.87±1.03 |  |
| NJ30 | 22.09±3.84 | 31.25±2.20 | 33.55±3.91 | 2.53±0.30 | 0.94±0.10 | 5.20±6.53 | 49.04±2.61 | 31.03±0.56 | 60.97±2.84 | 36.71±2.04 | 48.47±0.85 | 29.53±0.61 | 9.17±0.50 |  |
| NJ31 | 24.02±3.61 | 39.60±2.45 | 33.79±2.28 | 3.22±0.44 | 1.17±0.08 | 4.50±1.31 | 40.61±2.18 | 18.45±0.77 | 63.65±2.09 | 54.57±2.11 | 41.07±1.46 | 26.14±4.32 | 4.82±0.02 |  |
| NJ32 | 20.41±4.18 | 34.65±4.01 | 30.90±3.20 | 2.26±0.29 | 1.12±0.14 | 4.16±1.16 | 52.64±0.88 | 28.07±1.34 | 61.87±2.48 | 46.69±1.48 | 48.29±1.70 | 29.87±0.71 | 8.39±0.37 |  |
| NJ33 | 22.35±2.38 | 39.56±2.58 | 32.95±3.15 | 2.84±0.26 | 1.20±0.07 | 2.76±0.56 | 45.16±1.65 | 20.15±2.00 | 63.43±0.76 | 55.31±1.55 | 40.39±2.00 | 25.61±0.55 | 5.16±0.77 |  |
| YB1 | 19.65±5.22 | 32.39±2.78 | 32.39±4.00 | 1.92±0.32 | 1.00±0.10 | 4.27±1.24 | 56.75±2.64 | 21.78±1.46 | 77.60±1.08 | 61.76±4.00 | 46.41±0.67 | 36.09±1.02 | 7.43±0.64 |  |
| YB2 | 16.13±3.17 | 30.61±1.86 | 30.63±2.65 | 2.71±0.36 | 1.00±0.07 | 4.00±1.28 | 47.84±3.07 | 26.22±0.81 | 64.68±2.07 | 45.19±3.16 | 45.61±1.80 | 29.49±0.67 | 7.73±0.29 |  |
| YB3 | 21.52±5.25 | 36.18±3.23 | 32.55±3.68 | 2.64±0.43 | 1.11±0.10 | 4.70±2.07 | 46.38±3.11 | 21.86±1.88 | 61.24±2.44 | 52.88±2.07 | 43.89±1.07 | 26.88±1.00 | 5.89±0.18 |  |
| YB4 | 20.66±4.74 | 31.18±1.91 | 33.00±3.74 | 2.93±0.43 | 0.95±0.08 | 3.61±1.17 | 44.03±2.24 | 20.34±0.66 | 57.26±2.00 | 53.80±2.61 | 42.02±1.34 | 24.06±0.77 | 4.89±0.42 |  |
| YB5 | 14.19±2.77 | 29.67±2.12 | 28.20±3.29 | 1.90±0.29 | 1.06±0.12 | 3.57±1.29 | 47.49±1.48 | 21.79±0.71 | 66.17±3.19 | 54.09±1.99 | 48.01±2.35 | 31.79±0.91 | 6.93±0.41 |  |
| YB6 | 12.99±1.73 | 27.99±2.04 | 27.56±1.91 | 2.20±0.37 | 1.01±0.09 | 2.23±0.72 | 48.73±1.54 | 27.73±0.82 | 49.36±2.46 | 43.11±3.49 | 45.51±1.66 | 22.53±0.88 | 6.26±0.38 |  |
| YB7 | 24.16±3.49 | 32.96±2.07 | 34.60±2.54 | 2.96±0.30 | 0.95±0.07 | 3.40±1.14 | 40.70±1.29 | 26.53±1.22 | 66.39±2.33 | 34.91±1.08 | 56.34±0.82 | 37.41±1.02 | 9.94±0.61 |  |
| YB8 | 19.20±3.38 | 34.77±2.59 | 32.35±2.95 | 2.90±0.30 | 1.08±0.11 | 3.33±1.14 | 42.12±2.07 | 26.04±1.30 | 64.27±2.18 | 38.26±2.16 | 54.00±1.79 | 34.71±0.67 | 9.05±1.10 |  |
| YB9 | 31.34±3.86 | 36.33±1.20 | 37.50±2.74 | 3.46±0.34 | 0.97±0.06 | 2.77±0.36 | 40.83±3.46 | 18.26±1.59 | 42.68±1.79 | 55.26±4.70 | 41.77±1.80 | 17.80±0.43 | 3.25±0.61 |  |
| YB10 | 33.54±3.89 | 33.98±1.87 | 33.46±2.29 | 3.71±0.44 | 1.01±0.05 | 2.71±0.98 | 38.54±1.48 | 18.64±0.80 | 67.70±3.84 | 51.74±3.11 | 41.36±1.22 | 28.00±0.71 | 5.19±0.09 |  |
| YB11 | 22.08±3.45 | 34.73±1.77 | 33.97±2.30 | 2.34±0.32 | 1.02±0.06 | 4.37±1.16 | 52.03±0.87 | 28.09±1.66 | 54.09±0.58 | 45.99±2.12 | 48.50±1.74 | 26.23±0.41 | 7.37±0.48 |  |
| YB12 | 27.59±4.31 | 34.52±1.76 | 36.44±2.40 | 3.82±0.59 | 0.94±0.05 | 3.37±1.15 | 40.31±1.67 | 18.89±1.45 | 59.41±1.53 | 53.06±2.60 | 45.03±0.61 | 26.77±1.00 | 5.06±0.06 |  |
| YB13 | 18.23±2.80 | 29.46±1.51 | 32.40±1.99 | 2.57±0.25 | 0.91±0.05 | 5.90±1.44 | 44.15±2.18 | 31.46±0.67 | 74.58±1.19 | 28.74±1.03 | 49.63±0.88 | 37.02±0.77 | 11.64±0.33 |  |
| YB14 | 16.39±2.32 | 29.90±1.44 | 30.24±2.36 | 2.13±0.45 | 0.99±0.06 | 4.29±1.38 | 53.81±1.99 | 22.21±2.19 | 54.32±2.70 | 58.70±2.89 | 39.41±1.20 | 21.41±0.46 | 4.76±0.82 |  |
| YB15 | 15.69±2.91 | 29.05±1.89 | 28.75±2.74 | 2.39±0.34 | 1.01±0.07 | 2.70±0.79 | 44.62±1.81 | 19.84±0.48 | 55.85±2.64 | 55.59±2.08 | 42.66±0.16 | 23.81±0.55 | 4.72±0.44 |  |
| YB16 | 22.17±3.62 | 39.90±2.50 | 31.93±2.77 | 3.56±0.46 | 1.25±0.13 | 3.85±1.21 | 39.59±2.67 | 21.86±2.46 | 61.94±2.19 | 44.76±2.46 | 46.07±0.80 | 28.54±0.30 | 6.24±0.71 |  |
| YB17 | 16.59±2.15 | 28.79±1.68 | 30.95±2.10 | 2.66±0.22 | 0.93±0.07 | 3.58±1.04 | 44.32±2.22 | 28.56±2.18 | 63.53±0.88 | 35.59±1.01 | 55.05±2.00 | 34.97±1.07 | 10.00±0.29 |  |
| YB18 | 21.49±3.07 | 33.83±2.14 | 32.92±2.33 | 2.33±0.25 | 1.03±0.09 | 4.50±1.20 | 48.54±2.84 | 29.68±1.46 | 68.34±1.50 | 38.84±0.82 | 52.92±1.89 | 36.16±0.64 | 10.73±1.02 |  |
| LZ1 | 19.09±1.75 | 34.61±1.52 | 32.16±2.16 | 2.63±0.20 | 1.08±0.08 | 4.91±1.11 | 46.47±3.19 | 26.44±0.49 | 65.48±2.44 | 42.92±1.55 | 45.40±0.88 | 29.70±0.72 | 7.90±0.33 |  |
| LZ2 | 18.63±2.23 | 29.94±1.54 | 31.48±2.21 | 2.67±0.42 | 0.95±0.08 | 3.70±0.97 | 43.61±2.04 | 25.05±1.72 | 73.88±3.08 | 42.56±2.07 | 50.51±1.46 | 37.38±0.84 | 9.41±0.46 |  |
| LZ3 | 20.20±3.36 | 39.62±4.48 | 30.82±2.28 | 2.80±0.26 | 1.28±0.16 | 4.33±1.33 | 45.30±2.18 | 27.68±0.49 | 69.34±3.11 | 38.93±1.60 | 44.96±1.87 | 31.19±0.49 | 8.64±0.58 |  |
| LZ4 | 25.16±3.59 | 36.86±2.66 | 34.94±3.01 | 2.91±0.30 | 1.06±0.12 | 3.66±0.78 | 41.64±1.67 | 16.89±1.45 | 46.09±1.66 | 59.43±3.61 | 33.87±0.23 | 15.61±0.16 | 2.64±0.07 |  |
| LZ5 | 19.97±3.95 | 34.13±1.86 | 31.63±2.28 | 2.58±0.42 | 1.08±0.05 | 3.93±1.06 | 41.04±1.49 | 22.52±2.07 | 61.29±1.92 | 45.14±2.66 | 49.37±0.37 | 30.26±0.53 | 6.83±0.06 |  |
| LZ6 | 20.80±3.28 | 29.77±1.70 | 33.91±3.94 | 2.90±0.26 | 0.89±0.12 | 4.09±1.15 | 39.88±2.58 | 24.03±2.00 | 68.01±2.15 | 39.73±1.90 | 54.47±1.60 | 37.04±0.80 | 8.90±0.44 |  |
| LZ7 | 22.91±3.56 | 35.81±2.04 | 33.43±2.33 | 2.84±0.36 | 1.07±0.06 | 2.90±0.79 | 42.94±2.09 | 18.32±1.64 | 66.99±3.07 | 57.34±2.81 | 37.58±1.46 | 25.17±0.91 | 4.61±0.61 |  |
| YA1 | 22.24±4.17 | 35.49±2.40 | 34.79±2.32 | 2.99±0.32 | 1.02±0.05 | 5.02±1.34 | 46.28±1.49 | 24.92±2.75 | 74.28±2.63 | 46.25±2.05 | 45.73±2.01 | 33.94±0.63 | 8.44±1.06 |  |
| YA2 | 48.14±8.34 | 45.60±1.93 | 43.94±2.50 | 4.49±0.62 | 1.04±0.05 | 8.80±2.35 | 39.44±1.34 | 18.49±2.05 | 67.22±4.95 | 53.06±3.88 | 51.73±0.63 | 34.77±1.32 | 6.42±0.14 |  |
| YA3 | 20.03±4.41 | 39.07±3.46 | 33.06±2.85 | 3.79±0.47 | 1.18±0.09 | 2.66±1.23 | 30.47±2.75 | 15.23±1.61 | 62.88±2.52 | 49.84±3.11 | 46.93±1.06 | 29.50±0.49 | 4.49±0.23 |  |
| YA4 | 30.39±5.18 | 39.65±3.29 | 38.91±2.07 | 3.76±0.46 | 1.02±0.09 | 4.33±1.15 | 41.10±2.77 | 21.42±2.24 | 72.84±5.07 | 47.87±4.00 | 55.14±2.05 | 40.19±2.61 | 8.64±1.18 |  |
| YA5 | 11.18±2.56 | 28.87±2.51 | 29.15±2.58 | 2.66±0.58 | 1.00±0.12 | 1.76±0.70 | 41.75±3.64 | 21.73±2.83 | 68.89±1.76 | 47.56±3.49 | 51.33±1.24 | 35.36±0.70 | 7.69±0.87 |  |
| YA6 | 7.70±2.10 | 25.35±4.14 | 23.03±4.40 | 2.90±0.55 | 1.12±0.14 | 1.73±0.87 | 39.57±1.88 | 25.20±1.51 | 76.80±1.80 | 36.35±1.41 | 59.48±1.10 | 45.68±0.81 | 11.51±0.16 |  |
| YA7 | 41.75±7.96 | 40.18±3.98 | 44.51±2.86 | 4.30±0.49 | 0.90±0.07 | 9.64±2.92 | 42.30±1.73 | 21.62±1.62 | 75.67±2.30 | 48.93±2.40 | 54.60±0.73 | 41.30±0.60 | 8.93±0.62 |  |
| YA8 | 22.65±3.47 | 33.28±1.88 | 35.89±1.84 | 4.16±0.54 | 0.93±0.04 | 5.06±1.19 | 41.42±2.51 | 24.90±2.20 | 74.81±0.97 | 39.81±2.34 | 57.87±0.92 | 43.29±0.67 | 10.78±0.22 |  |
| YA9 | 20.87±2.35 | 35.17±1.38 | 33.60±1.58 | 2.28±0.30 | 1.05±0.04 | 6.04±1.60 | 46.19±0.55 | 34.30±1.42 | 74.39±1.42 | 25.73±2.41 | 57.39±0.28 | 42.70±0.21 | 14.64±0.07 |  |
| YA10 | 27.39±5.79 | 43.22±3.49 | 36.35±2.88 | 3.81±0.46 | 1.19±0.10 | 4.96±1.64 | 44.95±2.45 | 24.13±1.27 | 65.90±3.07 | 46.28±2.12 | 51.55±0.38 | 33.96±1.45 | 8.19±0.18 |  |
| YA11 | 27.36±2.59 | 36.28±2.12 | 37.74±2.69 | 4.26±0.44 | 0.97±0.09 | 7.48±1.93 | 37.24±4.03 | 23.63±3.06 | 62.93±2.69 | 36.28±1.18 | 53.24±2.22 | 33.53±2.21 | 7.88±0.33 |  |
| YA12 | 31.27±5.11 | 41.25±2.62 | 38.95±2.00 | 3.52±0.65 | 1.06±0.08 | 5.71±1.31 | 47.74±3.03 | 24.29±2.43 | 65.31±3.71 | 49.40±1.40 | 46.58±2.35 | 30.39±0.74 | 7.36±1.13 |  |
| YA13 | 34.10±5.67 | 37.38±2.36 | 41.20±2.48 | 4.27±0.66 | 0.91±0.04 | 4.89±1.58 | 38.44±1.93 | 20.23±1.22 | 67.85±1.58 | 47.24±4.43 | 50.12±1.05 | 34.00±0.37 | 6.88±0.26 |  |
| YA14 | 27.04±5.11 | 36.77±2.86 | 38.86±2.47 | 4.16±0.65 | 0.95±0.04 | 5.74±2.25 | 41.76±1.09 | 19.71±2.66 | 70.01±2.30 | 52.85±3.68 | 53.59±0.17 | 37.52±0.13 | 7.40±0.99 |  |
| YA15 | 23.98±3.26 | 40.67±2.66 | 35.60±1.77 | 4.04±0.56 | 1.14±0.09 | 2.84±0.94 | 34.26±3.29 | 16.34±1.39 | 63.13±5.11 | 52.00±2.96 | 50.17±2.30 | 31.65±0.83 | 5.17±0.04 |  |
| YA16 | 31.30±5.07 | 35.03±2.01 | 40.83±3.33 | 4.78±0.58 | 0.86±0.05 | 6.30±1.92 | 34.65±1.48 | 19.15±1.50 | 60.03±5.12 | 44.74±3.30 | 54.96±2.77 | 33.06±3.52 | 6.35±0.83 |  |
| YA17 | 24.24±4.18 | 34.32±2.01 | 36.97±2.14 | 3.76±0.66 | 0.93±0.07 | 5.00±1.35 | 41.41±3.43 | 22.04±3.25 | 67.82±1.63 | 46.90±3.01 | 52.41±1.79 | 35.54±1.19 | 7.83±1.04 |  |
| YA18 | 17.40±2.58 | 31.13±2.43 | 32.86±2.82 | 3.23±0.47 | 0.95±0.04 | 4.18±1.10 | 42.05±1.18 | 22.75±4.17 | 71.82±1.86 | 45.83±1.53 | 52.64±0.94 | 37.80±0.81 | 8.62±1.70 |  |
| YA19 | 29.15±6.25 | 36.05±2.03 | 38.98±2.93 | 3.45±0.49 | 0.93±0.05 | 5.50±1.72 | 45.89±2.37 | 28.05±2.51 | 70.84±1.51 | 38.61±0.77 | 56.32±1.60 | 39.90±1.14 | 11.20±0.79 |  |
| YA20 | 21.48±2.92 | 32.87±3.47 | 34.88±1.89 | 3.29±0.60 | 0.94±0.06 | 4.42±0.90 | 45.48±2.76 | 29.03±2.52 | 76.14±1.73 | 36.23±2.43 | 56.95±3.93 | 43.12±3.59 | 12.83±0.32 |  |
| YA21 | 22.57±3.48 | 33.56±1.50 | 35.51±2.12 | 3.26±0.37 | 0.95±0.06 | 3.89±1.52 | 36.98±3.20 | 17.31±2.43 | 66.98±3.05 | 54.69±3.25 | 50.65±1.21 | 33.95±1.59 | 5.90±0.91 |  |
| YA22 | 27.00±4.59 | 44.03±3.87 | 36.49±2.25 | 2.61±0.53 | 1.21±0.10 | 3.00±0.80 | 52.66±4.48 | 24.30±1.51 | 67.53±4.76 | 54.10±2.85 | 52.70±0.68 | 35.58±2.16 | 8.71±1.86 |  |
| YA23 | 23.37±4.21 | 33.83±3.65 | 37.31±2.24 | 3.56±0.40 | 0.91±0.09 | 6.61±2.02 | 42.16±3.24 | 26.86±2.48 | 80.44±1.53 | 36.35±1.03 | 58.86±0.53 | 47.35±0.43 | 12.72±0.11 |  |
| YA24 | 22.69±4.41 | 32.86±2.50 | 36.37±2.48 | 3.76±0.56 | 0.90±0.04 | 5.06±2.01 | 34.09±3.00 | 19.71±1.07 | 68.67±1.29 | 41.87±1.14 | 57.96±2.52 | 39.80±1.72 | 7.84±0.34 |  |
| YA25 | 20.10±4.61 | 32.07±2.51 | 33.52±3.59 | 3.56±0.42 | 0.96±0.08 | 3.39±1.08 | 32.45±3.00 | 18.30±1.94 | 68.37±4.90 | 43.55±3.99 | 56.71±1.20 | 39.70±0.65 | 7.80±0.68 |  |
| YA26 | 19.69±2.88 | 35.99±1.80 | 33.87±2.32 | 2.93±0.42 | 1.07±0.07 | 5.63±1.35 | 49.28±4.40 | 23.84±2.88 | 75.30±1.40 | 51.73±1.68 | 49.50±0.81 | 37.28±1.19 | 8.91±1.04 |  |
| YA27 | 19.82±2.63 | 35.26±3.36 | 33.45±3.53 | 3.17±0.52 | 1.06±0.09 | 3.80±0.99 | 46.48±1.11 | 19.79±2.11 | 72.00±2.48 | 57.48±3.90 | 53.03±1.82 | 38.20±2.11 | 7.59±1.13 |  |
| YA28 | 21.50±2.72 | 30.83±1.56 | 35.88±2.18 | 3.14±0.56 | 0.86±0.07 | 7.47±1.69 | 47.77±4.30 | 22.77±1.59 | 70.26±1.65 | 52.06±4.07 | 55.75±2.10 | 39.17±1.49 | 8.92±0.34 |  |
| YA29 | 31.46±5.79 | 40.75±3.29 | 38.14±2.47 | 4.01±0.48 | 1.07±0.05 | 3.96±1.29 | 42.97±1.64 | 21.13±1.79 | 65.47±2.50 | 50.90±2.59 | 46.03±2.90 | 30.14±1.98 | 6.37±0.67 |  |
| YA30 | 30.37±5.48 | 37.05±1.57 | 40.58±3.91 | 4.08±1.07 | 0.92±0.12 | 6.13±1.51 | 40.73±5.68 | 28.56±1.78 | 72.78±7.51 | 28.88±9.43 | 55.88±1.91 | 40.63±0.51 | 11.60±0.14 |  |
| YA31 | 22.52±3.50 | 34.17±2.38 | 35.10±1.93 | 3.45±0.57 | 0.97±0.06 | 4.76±1.76 | 41.31±1.71 | 20.05±0.76 | 63.40±3.12 | 51.39±2.60 | 52.22±1.62 | 33.12±1.43 | 6.65±0.43 |  |
| YA32 | 24.96±3.08 | 36.99±2.08 | 36.14±1.57 | 4.51±0.60 | 1.02±0.05 | 3.75±0.89 | 32.49±1.97 | 15.82±0.48 | 68.00±1.76 | 51.22±1.95 | 47.62±3.35 | 32.39±2.37 | 5.13±0.44 |  |
| YA33 | 25.55±3.80 | 33.67±2.24 | 37.83±1.76 | 2.68±0.25 | 0.89±0.04 | 6.41±1.66 | 50.12±1.54 | 31.80±1.55 | 79.32±1.30 | 36.57±1.82 | 57.22±2.46 | 45.39±1.97 | 14.45±1.07 |  |
| YA34 | 24.52±4.39 | 33.83±2.63 | 38.05±2.55 | 3.69±0.51 | 0.89±0.07 | 3.91±1.48 | 37.82±2.31 | 23.75±1.25 | 70.00±2.35 | 37.08±3.76 | 56.44±2.28 | 39.54±2.60 | 9.39±0.67 |  |
| YA35 | 26.23±5.29 | 39.84±3.73 | 37.38±3.24 | 2.85±0.50 | 1.07±0.07 | 5.09±1.64 | 49.49±2.90 | 27.58±3.69 | 69.31±2.96 | 44.32±1.00 | 58.34±1.62 | 40.41±1.22 | 11.17±1.55 |  |
| YA36 | 14.43±2.23 | 29.93±1.93 | 30.29±1.78 | 3.41±0.46 | 0.99±0.04 | 2.81±0.60 | 36.75±2.89 | 19.69±1.13 | 74.35±1.38 | 46.63±5.80 | 49.16±0.66 | 36.55±0.53 | 7.19±0.78 |  |
| YA37 | 29.81±5.46 | 42.09±3.45 | 37.41±2.46 | 3.90±0.56 | 1.13±0.06 | 4.22±0.85 | 43.67±2.44 | 22.40±3.31 | 73.07±1.10 | 48.85±4.04 | 49.59±0.52 | 36.24±0.36 | 8.11±1.14 |  |
| YA38 | 26.87±5.48 | 39.66±4.15 | 37.75±2.06 | 3.74±0.51 | 1.05±0.08 | 4.00±1.44 | 38.61±3.21 | 14.54±1.06 | 64.86±1.84 | 62.16±3.81 | 39.97±2.00 | 25.92±1.24 | 3.77±0.19 |  |
| YA39 | 14.41±3.35 | 35.39±2.29 | 29.55±3.06 | 2.92±0.54 | 1.21±0.13 | 2.18±1.01 | 43.72±4.40 | 27.18±4.23 | 74.97±1.04 | 38.11±3.80 | 56.37±1.83 | 42.27±1.69 | 11.43±1.31 |  |
| YA40 | 31.56±3.80 | 41.93±2.75 | 39.24±1.92 | 4.20±0.49 | 1.07±0.07 | 5.03±1.29 | 38.72±3.22 | 21.84±2.83 | 68.91±1.75 | 43.69±4.02 | 56.83±2.28 | 39.17±1.79 | 8.59±1.25 |  |
| YA41 | 29.56±3.42 | 38.08±3.47 | 40.53±1.95 | 4.05±0.39 | 0.94±0.09 | 6.46±1.90 | 38.66±1.64 | 21.51±1.36 | 73.25±2.51 | 44.33±3.34 | 55.68±1.06 | 40.77±1.03 | 8.76±0.24 |  |
| YA42 | 28.09±3.56 | 37.91±3.22 | 37.17±2.94 | 4.21±0.64 | 1.02±0.07 | 5.22±1.11 | 34.96±3.03 | 19.81±1.66 | 73.02±1.46 | 43.63±2.00 | 52.56±2.37 | 38.40±2.36 | 7.63±0.94 |  |
| YA43 | 26.52±3.37 | 35.41±2.26 | 38.70±2.24 | 4.07±0.77 | 0.92±0.09 | 3.40±0.94 | 38.72±4.32 | 19.97±3.48 | 63.06±3.16 | 48.67±4.46 | 40.74±1.74 | 25.69±1.11 | 5.14±0.97 |  |
| YA44 | 13.58±2.07 | 29.32±2.37 | 30.84±2.36 | 3.01±0.39 | 0.95±0.08 | 3.82±0.91 | 43.91±2.84 | 20.30±2.24 | 63.17±3.60 | 53.69±5.19 | 56.38±1.54 | 35.60±1.23 | 7.24±0.67 |  |
| YA45 | 28.96±5.63 | 36.90±3.48 | 39.36±2.58 | 3.77±0.65 | 0.94±0.07 | 3.08±1.12 | 43.80±1.97 | 16.97±2.86 | 63.91±5.84 | 61.10±5.65 | 44.63±0.64 | 28.53±2.17 | 4.88±0.95 |  |
| YA46 | 14.58±2.33 | 30.27±2.20 | 30.15±2.04 | 3.10±0.47 | 1.01±0.06 | 3.96±1.03 | 44.28±1.59 | 26.29±2.46 | 71.36±1.30 | 39.16±2.40 | 57.22±2.40 | 40.81±1.86 | 10.59±1.45 |  |
| YA47 | 16.01±3.09 | 30.31±1.98 | 32.60±2.40 | 2.45±0.45 | 0.93±0.05 | 3.72±1.02 | 51.47±1.12 | 29.60±2.99 | 73.94±2.15 | 41.71±1.07 | 54.20±0.80 | 40.08±1.22 | 11.89±1.50 |  |
| YA48 | 14.60±3.19 | 32.20±3.11 | 28.86±2.38 | 3.17±0.64 | 1.12±0.11 | 3.82±1.51 | 38.13±3.92 | 22.74±2.41 | 69.80±3.03 | 40.35±1.61 | 55.95±2.29 | 39.08±2.30 | 8.92±1.25 |  |
| YA49 | 22.66±3.67 | 38.82±3.20 | 34.34±1.94 | 3.93±0.46 | 1.13±0.09 | 5.45±1.50 | 41.86±2.38 | 15.60±1.98 | 55.40±2.61 | 63.54±4.79 | 46.76±1.95 | 25.95±1.26 | 4.12±0.39 |  |
| YA50 | 36.74±7.25 | 41.11±2.58 | 40.71±2.63 | 4.36±0.63 | 1.01±0.06 | 4.75±1.13 | 42.49±2.02 | 20.53±1.22 | 70.17±1.18 | 52.16±1.15 | 48.79±1.02 | 34.23±0.76 | 7.02±0.23 |  |
| YA51 | 20.04±3.14 | 31.22±1.89 | 35.31±1.78 | 3.01±0.66 | 0.88±0.05 | 4.00±1.02 | 46.05±0.72 | 25.95±1.65 | 73.94±0.73 | 43.68±3.01 | 50.73±0.62 | 37.35±0.60 | 9.30±1.27 |  |
| YA52 | 25.11±1.99 | 35.47±1.78 | 37.10±1.43 | 3.42±0.52 | 0.96±0.06 | 3.64±1.01 | 43.57±3.64 | 21.98±1.52 | 65.97±2.40 | 50.29±2.37 | 48.65±0.65 | 32.09±0.41 | 7.06±1.34 |  |
| YA53 | 21.77±3.52 | 35.81±2.47 | 35.87±2.53 | 2.77±0.48 | 1.00±0.09 | 5.08±1.23 | 48.99±3.41 | 28.05±1.85 | 71.34±1.72 | 42.50±3.12 | 56.40±0.51 | 40.23±1.04 | 11.30±0.90 |  |
| YA54 | 36.49±4.36 | 37.92±3.03 | 41.28±2.72 | 3.69±0.53 | 0.92±0.08 | 5.67±1.37 | 41.21±1.49 | 20.69±0.82 | 63.58±5.10 | 49.75±2.21 | 54.66±3.16 | 34.71±1.24 | 7.18±0.13 |  |
| YA55 | 22.81±2.72 | 31.85±1.66 | 36.89±1.51 | 2.79±0.27 | 0.86±0.04 | 6.80±1.50 | 41.28±4.76 | 22.49±2.47 | 64.65±2.61 | 45.35±4.12 | 50.91±2.46 | 32.93±1.97 | 7.41±0.51 |  |
| YA56 | 12.56±2.58 | 28.73±3.28 | 29.81±3.36 | 4.14±1.23 | 0.96±0.06 | 4.52±1.47 | 34.06±2.44 | 19.42±3.83 | 66.73±2.59 | 39.27±5.88 | 62.08±1.31 | 41.44±2.14 | 8.01±0.48 |  |
| YA57 | 24.17±2.32 | 38.79±2.67 | 36.06±1.50 | 3.16±0.40 | 1.08±0.06 | 4.71±1.45 | 45.68±1.39 | 21.80±0.54 | 70.50±2.03 | 52.25±1.78 | 52.77±2.35 | 37.17±0.86 | 8.10±0.23 |  |
| YA58 | 22.13±2.14 | 33.48±2.11 | 35.95±1.47 | 3.13±0.71 | 0.93±0.07 | 4.98±1.42 | 44.97±2.56 | 18.71±1.03 | 59.43±1.75 | 58.26±3.70 | 40.62±2.43 | 24.13±1.35 | 4.52±0.34 |  |
| YA59 | 17.32±2.49 | 32.35±1.65 | 32.35±1.77 | 2.95±0.45 | 1.00±0.06 | 4.92±1.56 | 47.60±4.52 | 29.01±0.53 | 74.36±2.40 | 38.91±2.73 | 53.47±2.55 | 39.76±1.80 | 11.56±1.44 |  |
| YA60 | 18.54±3.52 | 37.62±2.72 | 32.81±2.32 | 3.30±0.37 | 1.15±0.13 | 2.74±0.94 | 35.93±1.48 | 20.06±1.64 | 71.81±2.92 | 44.30±1.01 | 56.86±2.32 | 40.86±2.51 | 8.26±1.85 |  |
| DZ1 | 22.08±2.74 | 35.88±2.31 | 35.97±2.21 | 3.36±0.58 | 1.00±0.09 | 2.44±0.87 | 42.23±1.87 | 20.54±1.94 | 64.75±4.56 | 51.42±2.94 | 48.10±1.21 | 31.12±1.70 | 6.41±0.81 |  |
| DZ2 | 21.60±2.75 | 36.10±1.90 | 34.35±2.00 | 3.80±0.46 | 1.05±0.08 | 4.86±1.35 | 37.33±1.88 | 22.93±1.50 | 76.55±0.77 | 38.58±2.05 | 61.76±1.87 | 47.28±1.57 | 10.85±0.61 |  |
| DZ3 | 26.23±4.60 | 37.04±2.30 | 36.58±3.47 | 2.93±0.45 | 1.02±0.11 | 3.97±1.13 | 52.23±0.99 | 25.51±2.22 | 66.63±1.65 | 42.04±2.15 | 52.71±1.86 | 35.16±1.06 | 10.93±0.50 |  |
| DZ4 | 16.78±3.13 | 32.79±3.03 | 32.69±2.17 | 2.30±0.29 | 1.01±0.09 | 2.76±1.21 | 55.15±1.44 | 30.46±1.39 | 61.87±2.77 | 44.73±2.90 | 49.38±0.51 | 30.55±1.32 | 9.32±0.72 |  |
| DZ5 | 21.29±4.12 | 36.55±2.97 | 32.25±2.84 | 4.13±0.87 | 1.14±0.13 | 2.60±0.84 | 30.68±3.61 | 15.90±1.67 | 72.40±1.39 | 48.04±3.05 | 43.28±1.75 | 31.33±1.26 | 4.98±0.48 |  |
| DZ6 | 19.78±4.91 | 38.24±3.56 | 33.92±3.14 | 3.08±0.39 | 1.13±0.08 | 3.50±1.25 | 37.20±0.58 | 23.28±0.34 | 71.89±2.92 | 37.41±1.42 | 44.54±7.68 | 31.82±5.83 | 7.41±1.35 |  |
| DZ7 | 22.47±6.05 | 34.04±3.50 | 35.44±3.51 | 4.55±0.78 | 0.97±0.12 | 3.63±1.31 | 36.10±2.96 | 20.93±2.09 | 68.43±3.13 | 41.98±3.99 | 52.70±2.93 | 36.06±1.87 | 7.56±0.70 |  |
| DZ8 | 32.54±5.05 | 38.84±2.37 | 38.56±3.01 | 3.86±0.43 | 1.01±0.07 | 3.82±1.07 | 40.56±0.79 | 20.74±1.17 | 78.65±1.42 | 48.86±2.91 | 51.33±2.08 | 40.37±1.53 | 8.37±0.28 |  |
| DZ9 | 16.66±3.32 | 34.97±2.35 | 29.32±2.35 | 3.16±0.58 | 1.20±0.10 | 2.48±0.89 | 38.80±2.32 | 24.45±3.11 | 73.15±2.53 | 37.09±5.86 | 50.82±0.66 | 37.17±0.48 | 9.09±0.12 |  |
| Max | 49.03 | 46.11 | 45.47 | 4.78 | 1.41 | 9.64 | 61.8 | 36.66 | 80.44 | 69.19 | 62.08 | 47.35 | 14.64 |  |
| Min | 7.70 | 25.35 | 23.03 | 1.51 | 0.71 | 1.73 | 25.49 | 14.54 | 42.68 | 25.73 | 18.58 | 8.81 | 1.45 |  |
| Mean | 24.35 | 35.76 | 35.12 | 3.11 | 1.02 | 4.62 | 44.55 | 23.64 | 65.54 | 46.7 | 48.66 | 32.27 | 7.79 |  |
| SD | 7.24 | 4.05 | 3.89 | 0.67 | 0.10 | 1.55 | 6.44 | 4.74 | 7.69 | 8 | 7.28 | 7.45 | 2.73 |  |
| Median | 22.94 | 35.49 | 35.09 | 2.93 | 1.01 | 4.50 | 44.32 | 22.81 | 66.85 | 46.25 | 49.59 | 33.34 | 7.69 |  |
| CV (%) | 29.72 | 11.32 | 11.07 | 21.42 | 10.22 | 33.57 | 14.46 | 20.07 | 11.74 | 17.14 | 14.97 | 23.10 | 35.05 |  |
| H′ | 1.98 | 2.06 | 2.06 | 2.06 | 1.99 | 2.01 | 2.07 | 2.06 | 1.96 | 2.04 | 1.98 | 2.03 | 2.06 |  |

Note: The values reported the mean of two years (2020 and 2021), and results show as mean ± SD.

**TABLE S3.** Variety number of 32 quantitative characteristics of each group.

| Groups | Variety number |
| --- | --- |
| I | ZG1, ZG2, ZG4, ZG5, ZG9, ZG10, ZG12, ZG16, NJ6, NJ7, NJ11, NJ15, NJ17, NJ18, NJ20, NJ22, NJ23, NJ25, NJ26, NJ27, NJ29, YB1, YB2, YB4, YB5, YB11, YB12, YB13, LZ3, LZ4, LZ5, LZ6, LZ7 |
| II | NJ1, YB14, YB15 |
| III | ZG3, ZG6, ZG8, ZG11, ZG13, NJ2, NJ3, NJ4, NJ8, NJ9, NJ10, NJ12, NJ14, NJ16, NJ19, NJ21, NJ24, NJ30, NJ31, NJ32, YB3, YB6, YB9, YB16, YB17, YB18, LZ1, LZ2 |
| IV | ZG7, ZG14, ZG15, NJ13, NJ28, NJ33, YB7, YB8, YB10 |
| V | NJ5, YA1, YA2, YA3, YA5, YA6, YA7, YA8, YA9, YA10, YA11, YA14, YA15, YA16, YA17, YA18, YA19, YA20, YA21, YA22, YA23, YA24, YA25, YA26, YA27, YA28, YA29, YA32, YA33, YA34, YA36, YA38, YA39, YA40, YA41, YA44, YA45, YA47, YA48, YA49, YA50, YA51, YA52, YA53, YA54, YA55, YA56, YA59, YA60, DZ2, DZ4, DZ5, DZ7, DZ8, DZ9 |
| VI | YA4, YA12, YA13, YA30, YA31, YA35, YA37, YA42, YA43, YA46, YA57, YA58, DZ1, DZ3, DZ6 |

**TABLE S4.** Mean value of 32 quantitative characteristics of each group

| Traits | Groups | | | | | |
| --- | --- | --- | --- | --- | --- | --- |
|  | I | II | III | IV | V | VI |
| Leaf length | 60.30 | 64.28 | 65.71 | 74.26 | 68.37 | 75.49 |
| Leaf width | 26.35 | 28.08 | 31.81 | 35.40 | 34.46 | 39.15 |
| Leaf area | 1065.00 | 1205.14 | 1400.81 | 1763.19 | 1585.28 | 1978.73 |
| Leaf shape index | 2.33 | 2.30 | 2.08 | 2.13 | 2.01 | 1.95 |
| Number of petals | 7.23 | 7.22 | 7.24 | 7.59 | 7.18 | 7.33 |
| Number of sepals | 7.48 | 7.22 | 7.39 | 7.88 | 5.81 | 5.73 |
| Number of stigmas | 3.42 | 3.06 | 3.30 | 3.37 | 3.01 | 3.20 |
| Flower crown | 60.84 | 60.50 | 61.40 | 65.23 | 57.11 | 56.90 |
| Petal length | 29.17 | 28.93 | 28.87 | 30.44 | 28.60 | 28.86 |
| Fruit weight | 23.57 | 18.96 | 25.12 | 27.55 | 23.78 | 25.88 |
| Fruit height | 35.24 | 31.45 | 36.28 | 36.27 | 35.54 | 37.23 |
| Fruit diameter | 34.12 | 31.43 | 34.73 | 35.71 | 35.54 | 36.94 |
| Peel thickness | 2.68 | 2.30 | 2.74 | 2.91 | 3.51 | 3.52 |
| Shape index of fruit | 1.04 | 1.00 | 1.05 | 1.02 | 1.01 | 1.01 |
| Number of seeds | 4.64 | 4.27 | 4.82 | 4.16 | 4.65 | 4.49 |
| Fresh seed rate | 46.63 | 51.53 | 47.46 | 46.68 | 41.56 | 42.85 |
| Dry seed rate | 24.34 | 21.81 | 25.36 | 25.65 | 22.36 | 22.70 |
| Kernel rate of dry seed | 62.45 | 55.60 | 61.11 | 65.77 | 69.39 | 68.35 |
| Moisture rate of fresh seed | 47.56 | 57.56 | 46.51 | 45.23 | 46.11 | 46.05 |
| Oil rate of kernel | 44.66 | 40.10 | 45.41 | 49.28 | 52.57 | 50.48 |
| Oil rate of dry seed | 28.38 | 22.29 | 27.99 | 32.45 | 36.67 | 34.62 |
| Oil rate of fresh fruit | 7.21 | 4.85 | 7.29 | 8.50 | 8.35 | 8.08 |
| Free acidity | 0.45 | 0.32 | 0.42 | 0.32 | 0.45 | 0.46 |
| Peroxide value | 0.05 | 0.06 | 0.05 | 0.04 | 0.02 | 0.02 |
| Palmitic acid | 11.80 | 12.22 | 12.02 | 11.33 | 11.14 | 10.83 |
| Stearic acid | 9.24 | 12.49 | 9.91 | 8.25 | 8.26 | 8.65 |
| Oleic acid | 76.40 | 73.29 | 75.50 | 77.78 | 78.25 | 78.36 |
| Linoleic acid | 2.21 | 1.64 | 2.25 | 2.35 | 2.06 | 1.89 |
| Eicosenoic acid | 0.35 | 0.36 | 0.32 | 0.29 | 0.29 | 0.27 |
| Squalene | 146.70 | 143.13 | 130.06 | 108.06 | 48.46 | 44.24 |
| α-Tocopherol | 262.28 | 608.33 | 269.32 | 272.66 | 53.03 | 42.54 |
| Sterol | 1434.58 | 1507.68 | 1146.16 | 995.48 | 5801.72 | 3964.67 |

**FIGURE S1** The Eigenvalue, Contribution rate (%) and Cumulative contribution rate (%) of from the PCA of 32 quantitative characters in the 143 wild *C. oleifera* accessions.


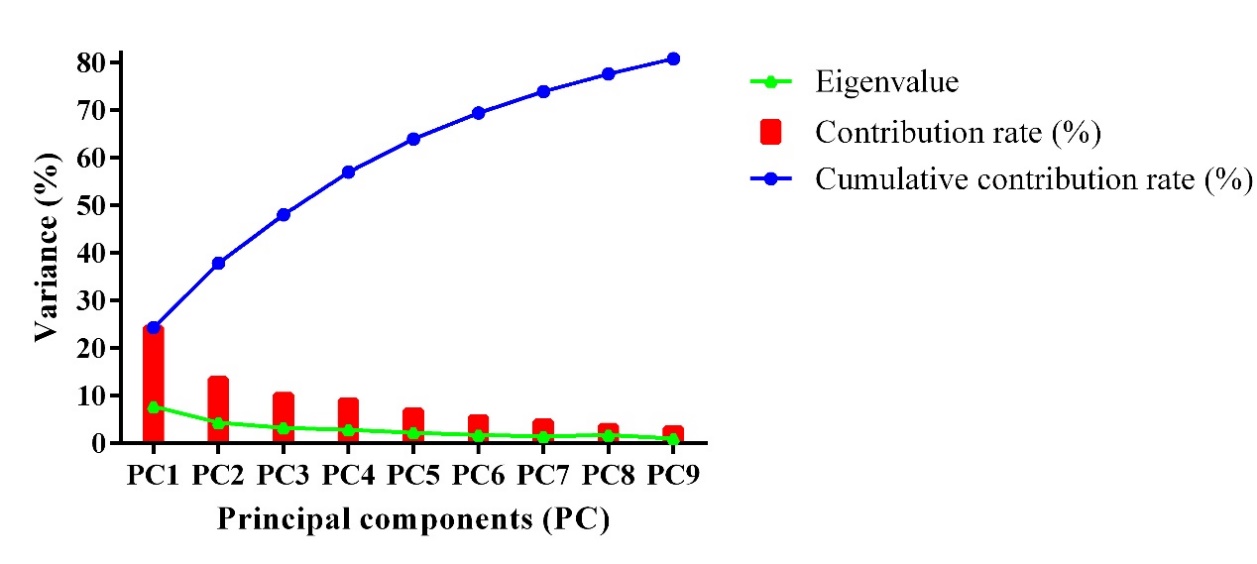

Supplement: Supplementary file 1 [file DataSheet_1.docx]
